# The translation initiation factor homolog *eif4e1c* regulates cardiomyocyte metabolism and proliferation during heart regeneration

**DOI:** 10.1242/dev.201376

**Published:** 2023-06-12

**Authors:** Anupama Rao, Baken Lyu, Ishrat Jahan, Anna Lubertozzi, Gao Zhou, Frank Tedeschi, Eckhard Jankowsky, Junsu Kang, Bryan Carstens, Kenneth D. Poss, Kedryn Baskin, Joseph Aaron Goldman

**Affiliations:** ^1^ Department of Biological Chemistry and Pharmacology, The Ohio State University Medical Center, Columbus, OH 43210, USA; ^2^ Department of Molecular Genetics, The Ohio State University, Columbus, OH 43210, USA; ^3^ Center for RNA Molecular Biology, Department of Biochemistry, School of Medicine, Case Western Reserve University, Cleveland, OH 44106 USA; ^4^ Department of Cell and Regenerative Biology, University of Wisconsin-Madison, Madison, WI 53705, USA; ^5^ Department of Evolution, Ecology, and Organismal Biology, The Ohio State University, Columbus, OH 43210, USA; ^6^ Department of Cell Biology, Duke Regeneration Center, Duke University School of Medicine, Durham, NC 27710, USA; ^7^ Department of Cell Biology and Physiology, The Ohio State University Medical Center, Columbus, OH 43210, USA

**Keywords:** Heart regeneration, mRNA translation, Zebrafish

## Abstract

The eIF4E family of translation initiation factors bind 5′ methylated caps and act as the limiting step for mRNA translation. The canonical eIF4E1A is required for cell viability, yet other related eIF4E families exist and are utilized in specific contexts or tissues. Here, we describe a family called Eif4e1c, for which we find roles during heart development and regeneration in zebrafish. The Eif4e1c family is present in all aquatic vertebrates but is lost in all terrestrial species. A core group of amino acids shared over 500 million years of evolution forms an interface along the protein surface, suggesting that Eif4e1c functions in a novel pathway. Deletion of *eif4e1c* in zebrafish caused growth deficits and impaired survival in juveniles. Mutants surviving to adulthood had fewer cardiomyocytes and reduced proliferative responses to cardiac injury. Ribosome profiling of mutant hearts demonstrated changes in translation efficiency of mRNA for genes known to regulate cardiomyocyte proliferation. Although *eif4e1c* is broadly expressed, its disruption had most notable impact on the heart and at juvenile stages. Our findings reveal context-dependent requirements for translation initiation regulators during heart regeneration.

## Teaser

**Summary:** The fish-specific translation initiation factor Eif4e1c, which is absent in terrestrial species, regulates growth and heart regeneration in zebrafish.

## INTRODUCTION

Zebrafish regenerate heart muscle after catastrophic injury. Fervent research is underway to discover molecules that regulate fundamental events in regeneration and have potential relevance to regenerative medicine. Changes in the abundance of dozens to thousands of mRNAs have been reported using both targeted approaches, such as *in situ* hybridization, and unbiased approaches based on RNA sequencing (RNAseq) (Goldman et al., 2017; Honkoop et al., 2018; Wu et al., 2016). However, reliance on mRNA as the sole measure of gene expression is an imperfect assumption at best, with only ∼40% of mRNA levels correlating with the amount of protein (Buccitelli and Selbach, 2020). Similarly, the abundance of mRNA and proteins are poorly interconnected during heart regeneration, suggesting an important role for post-transcriptional regulation (Ma et al., 2018). An alternative snapshot of expression levels was revealed by profiling of mRNA bound to a transgenic ribosomal subunit (Fang et al., 2013), which uncovered a role for JAK/STAT signaling in heart regeneration. However, few total transcripts were identified, likely owing to ribosome heterogeneities (Emmott et al., 2018; Genuth and Barna, 2018), the dependence on transgenesis, and/or limited starting material. Regulation of translation is an important mechanism that impacts gene expression (Kong and Lasko, 2012; Wang et al., 2020a) and is a central feature of physiological heart growth (Chorghade et al., 2017). How such regulation might impact heart regeneration, however, remains largely unexplored until now.

Pathways that initiate translation are diverse, yet the classical means is through loading of the ribosome onto start codons via recognition of 5′ methylated caps (Borden and Volpon, 2020). The canonical eIF4E1A major cap-binding protein is the rate-limiting factor for initiation of loading, and its expression levels help determine the translation of specific mRNA cohorts (Davis et al., 2019; Truitt et al., 2015). In mouse, eIF4E (hereafter referred to by the general family name eIF4E1A), encoded by the canonical *Eif4e1a* gene, is essential for development and viability (Sénéchal et al., 2021), limiting most mechanistic studies of its function to cell culture. However, studies of partial knockdowns and heterozygotes have shown *Eif4e1a* is generally found in excess, and at reduced levels improves resiliency to cancer in heterozygous mice (Graff et al., 2007; Truitt et al., 2015) and improved aging in adult worms (Syntichaki et al., 2007). Actively growing heart muscle in neonatal mice has increased eIF4E1A levels (Chen et al., 2022), but how eIF4E1A is regulated in a context of innate heart regeneration is unknown. Although the canonical eIF4E1A promotes translation, a second family of eIF4E1 homologs called eIF4E1B has been reported to have repressive functions in *Xenopus* and zebrafish (Minshall et al., 2007; Robalino et al., 2004). In fish, a third close relative of canonical eIF4E1A, called *eif4e1c*, has been reported (Joshi et al., 2005; Zhang et al., 2018), but its function and properties have not yet been described.

In a previous profiling study, we found that expression of the *eif4e1c* gene increases in zebrafish hearts during regeneration (Goldman et al., 2017). We show that *eif4e1c* represents a new and highly conserved family of eIF4E1 that is retained in aquatic vertebrate and lost in terrestrial animals. The evolutionary changes that maintain heart regeneration in some organisms and not others are an area of intense study, but with questions that still remain (Hirose et al., 2019; Lai et al., 2017; Stockdale et al., 2018; Wang et al., 2020b). Here, using CRISPR-generated mutants, we demonstrate that fish-specific *eif4e1c* is required for normal production of cardiomyocytes in zebrafish hearts during animal growth and injury-induced regeneration. Furthermore, our findings indicate that regulation of gene expression through translational mechanisms is a participating component of cardiogenesis.

## RESULTS AND DISCUSSION

### Eif4e1c is a family of translation initiation factors conserved in all aquatic vertebrates

**Fig. 1.**
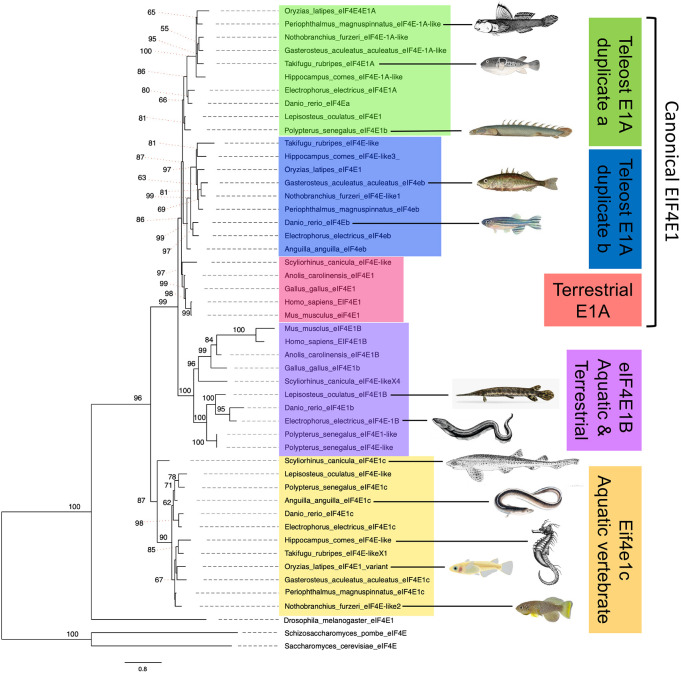
**Eif4e1c is both unique to and shared by all aquatic vertebrates.** Shown is a phylogeny of eIF4E1 orthologs from a sampling of the species considered (see Fig. S1 for full analysis). Eif4e1c is ancestral to the canonical eIF4E1A split from its variant, eIF4E1B. Terrestrial species have a canonical and eIF4E1B ortholog (pink and purple). All aquatic species have a canonical variant: eight species with a duplication (blue and green), two species retain only one variant of the duplication, and the *Scyliorhinus canicula* (small-spotted catshark) canonical clusters with terrestrial eIF4E1A. All 12 aquatic vertebrate retain an Eif4e1c family member. Shown are images of each of the aquatic vertebrate species to highlight the diversity considered. Only five of 12 aquatic vertebrates shown here retain an eIF4E1B variant. Estimates are made using maximum likelihood and the IQ-TREE. Nodes with bootstrap support <0.85 are marked with their respective values, all other nodes had support values of 0.85 or higher.

Zebrafish have two paralogs of the canonical *Eif4e1a*, *eif4ea* and *eif4eb*, that are likely a result of a whole genome duplication in teleost fish ∼380 million years ago (Taylor et al., 2001). Unlike the canonical *Eif4e1a*, zebrafish have only single copies of *eif4e1b* and *eif4e1c* (Joshi et al., 2005), raising the possibility that they are duplicates of one another. Given that the eIF4E1A and eIF4E1B families have distinct roles in translation, the evolutionary history of *eif4e1c* may help inform its function. To understand the relationship of *eif4e1c* with the two other eIF4E1 families, we undertook a phylogenetic analysis of eukaryotic eIF4E1 orthologs. Using the IQ-TREE web server (Trifinopoulos et al., 2016), we conducted a maximum likelihood analysis to estimate the phylogeny of 142 homologs from 53 species ranging from yeast to humans (Fig. S1A). Interestingly, Eif4E1c forms its own clade that is ancestral to the split of the Eif4e1a and Eif4e1b families (Fig. 1). A homolog of *eif4e1c* was found in all 37 aquatic vertebrates from this sampling, including non-teleosts, suggesting that the Eif4e1c family predates the teleost specific duplication event. Whereas the Eif4e1a and Eif4e1b families are shared by aquatic and terrestrial vertebrate species, the Eif4e1c clade is absent from terrestrial animals. We conclude that the Eif4e1c family must have been lost in an early ancestor of terrestrial vertebrates when coelacanth split from amphibians and is retained in all aquatic vertebrate fishes.

**Fig. 2.**
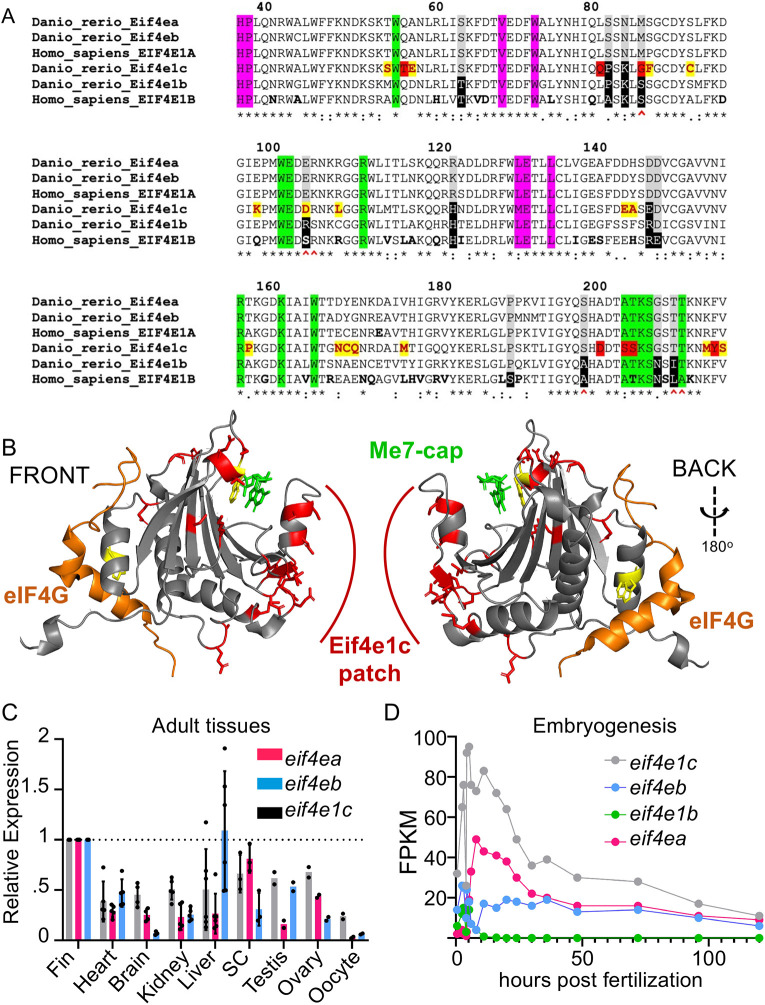
**Highly conserved Eif4e1c-specific amino acids form a novel patch along the surface of the protein.** (A) ClustalW sequence alignment of human versus fish Eif4E orthologs. Highlighted are universally conserved amino acids required for cap binding (green) and eIF4G recruitment (pink). Residues that distinguish EIF4E1A family members (gray) from EIF4E1B family members (black) are also highlighted. The six amino acids most responsible for strong cap binding are marked by red carets. The Eif4e1c family conserved residues are highlighted yellow with red letters. The seven residues that are identical in all EIF4E1A and EIF4E1B family orthologs throughout evolution but are uniquely changed in Eif4e1c family members are highlighted in red. Amino acids that diverge between mouse and human are bolded in the human sequence. (B) PyMOL rendition of zebrafish Eif4e1c mapped along the human structure of EIF4E1A bound to a methylated cap (green) and an EIF4G peptide (orange). The tryptophans required for both associations are conserved and highlighted in yellow. All Eif4e1c-specific residues are highlighted in red. (C) The abundance of each of the transcripts shown in the key were derived from RNAseq datasets from different adult tissues. Expression is determined relative to *mob4.* The FPKM for the three orthologs were similar in the fin. Values shown have been normalized to expression levels in the fin. Where error bars are presented, at least three biological replicates were used to determine the average and s.d. Only testis, ovary and oocyte had two replicates. Dots show individual data points. SC, spinal cord. (D) Time course of RNAseq of different developmental stages in embryos. Normalized sequencing reads for the four different EIF4E1 orthologs are shown.

To identify the sequence features that distinguish the Eif4e1c family, we performed ClustalW alignment between the four zebrafish homologs and their human orthologs (Fig. 2A). All six homologs share the essential amino acids for binding 5′ methylated caps (Fig. 2A, green) and assembly of the preinitiation complex with eIF4G (Fig. 2A, pink). In the 12 amino acids reported to distinguish eIF4E1A and eIF4E1B families (Kubacka et al., 2015), *eif4e1c* has substantially more similarity to eIF4E1A. For example, there are seven residues identical or similar to canonical eIF4E1A (Fig. 2A, gray boxes) and four residues identical to the eIF4E1B variant (black boxes); however, of the six residues that confer optimal binding affinity for caps (Fig. 2A, red carets), all six in *eif4e1c* share similarity with the canonical eIF4E1A. ClustalW alignment within the Eif4e1c family showed that the evolutionary conservation is striking (Fig. S1B). Shark and zebrafish Eif4e1c are 86% identical and 95% similar with only 8/186 substantial amino acid differences in the protein core over ∼500 million years of evolution. There are 23 amino acids that are invariant in almost all Eif4e1c family members (Fig. 2A, yellow boxes). Most of the 23 Eif4e1c residues are at locations in the protein where changes have been found and tolerated in other homologs from isolated species. Interestingly, seven of the 23 amino acids remain identical in all eIF4E1A and eIF4E1B orthologs throughout evolution, but are only different in the Eif4e1c family (red boxes). Thus, from amino acid sequence comparisons, we conclude that although the Eif4e1c family shares features with the canonical eIF4E1A in critical cap-binding residues, Eif4e1c is likely a unique homolog with both highly conserved and distinctive properties.

Crystal structures of eIF4E1A have been solved for several species and show that all homologs retain conserved features. To understand how the Eif4e1c distinctive residues may impact properties of the protein, we modeled zebrafish Eif4e1c over a crystal structure of human EIF4E1A bound to a capped GTP residue and a peptide from EIF4G (Grüner et al., 2016) using RoseTTAFold (Fig. 2B) (Baek et al., 2021). The canonical folds of the modeled zebrafish Eif4e1c protein are identical to what is found in the core of all eIF4E1 family members, with similar positioning of the two highly conserved tryptophans required for cap binding and preinitiation complex assembly (Fig. 2B, yellow). This mirrors what is seen in the AlphaFold prediction of the two molecules (Jumper et al., 2021). The 23 amino acids characteristic of the Eif4e1c family are positioned mainly along the protein surface in solvent exposed regions (Fig. 2B, red). A distinctive Eif4e1c-specific patch is found opposite the EIF4G-binding site and adjacent to the cap-binding region. Therefore, the highly conserved amino acids shared by the Eif4e1c family create a novel binding interface on the protein, suggesting that Eif4e1c interacts with unique partners.

### The *eif4e1c* gene is widely expressed in tissues and throughout development

Canonical eIF4E1A and eIF4E1B are known to have distinct expression patterns. For example, *eif4e1b* is only expressed in the gonads and skeletal muscle in adults and during the first 12 h post-fertilization (hpf) (Robalino et al., 2004). To determine whether *eif4e1c* is expressed in all tissues like eIF4E1A or is restricted to discrete organs or developmental stages like *eif4e1b*, we surveyed expression of *eif4e1c* in published RNAseq data sets (see Materials and Methods). *eif4ea*, *eif4eb* and *eif4e1c* genes were each expressed in every organ examined (Fig. 2C). Expression of *eif4e1c* was highest in the fin and in other tissues ranged from 38% to 67% of that total. These differences are likely within the typical RNAseq variability owing to batch effects, so we conclude that *eif4e1c* is widely expressed at similar levels throughout zebrafish organ systems. Although *eif4e1c* expression is widespread, whether it is confined to a particular cell types within organs is unclear. We analyzed a published single-cell (sc)RNAseq data set produced from adult zebrafish hearts that uncovered 15 different identifiable cell types (Hu et al., 2022). Transcripts for *eif4e1c*, and the canonical *eif4ea* and *eif4eb*, were detected within each of the 15 clusters, suggesting that all three transcripts are expressed in all cardiac cell types, including in cardiomyocytes (CMs) (Fig. S2). Thus, like its canonical orthologs, *eif4e1c* is broadly expressed in all organ systems and cell types examined.

The canonical homologs *eif4ea* and *eif4eb* were broadly expressed at similar levels as *eif4e1c* except for low expression of *eif4eb* in the brain (84% decrease) and *eif4ea* in the testis (74% decrease). Interestingly, *eif4e1c* was found to be the predominant homolog present in oocytes, 8.5-fold higher than *eif4ea* and 3.7-fold higher than *eif4eb* (Fig. 2C). High levels of maternal deposition suggest that *eif4e1c* may play a crucial role during embryogenesis. Using the EMBL expression atlas, we assessed expression levels of all eIF4E1 orthologs during a previously published time course across early zebrafish development (White et al., 2017). Zebrafish *eif4e1c* was the most highly expressed eIF4E1 at every time point during the first 5 days of development, during which most organ systems are formed (Fig. 2D). Expression levels of *eif4e1c* were roughly equal to the sum of both *eif4ea* and *eif4eb*, except during the first 12 hpf when *eif4e1c* was three to four times more abundant. During rapid growth stages, such as during embryogenesis, *eif4e1c* is the dominantly expressed cap-binding homolog but is expressed mostly at similar levels as the canonical factors in the adult.

### Deletion of the *eif4e1c* locus causes poor growth and survival to adulthood

**Fig. 3.**
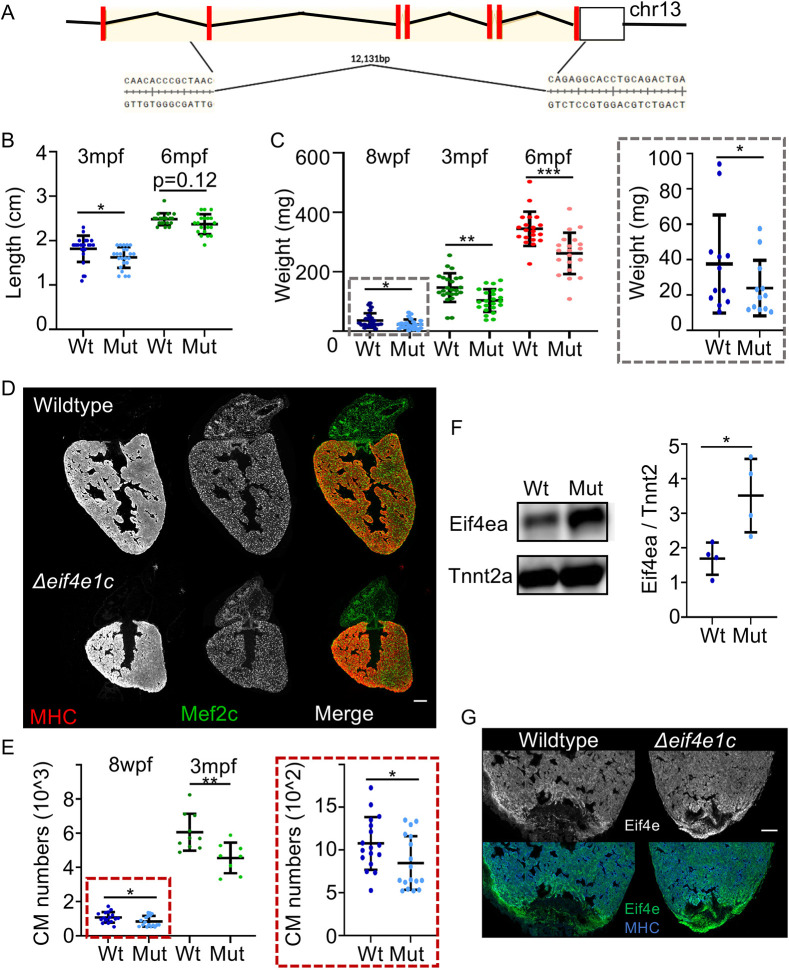
**Δ*eif4e1c* mutants have impaired growth and poor survival.** (A) Schematic of the CRISPR-mediated deletion of *eif4e1c*. Red rectangles represent coding exons, and the white box represents exons with untranslated regions. (B) Fish were grown together, genotyped at the indicated stage and immediately measured from jaw to the caudal fin bifurcation (3-month average wild type=1.82 cm, mutant=1.62 cm; Welch's *t*-test, *P*=0.0156; *n*=24 and 23, respectively; 6-month average wild type=2.48 cm, mutant=2.37 cm; Mann–Whitney, *P*=0.121; *n*=20 and 20, respectively). (C) After measuring length, fish were dried off as much as possible and weighed. The 8 wpf data (gray box) is shown on the right with a different scale for the *y*-axis. [8-week (blue) average wild type=35.75 mg, mutant=22.82 mg; Welch's *t*-test, *P=0.0392; *n*=24; 3-month (green) average wild type=146.5 mg, mutant=103.1 mg; Welch's *t*-test, **P=0.0014; *n*=24 and 23, respectively; 6-month average (red) wild type=344.6 mg, mutant=261.7 mg; Welch's *t*-test, ***P=0.0002; *n*=20). (D) Uninjured heart ventricles from wild-type and mutant fish were sectioned and stained for muscle using antibodies to myosin heavy chain (MHC) and the cardiac transcription factor Mef2c to identify CM nuclei. Scale bar: 100 µm. Note that adjacent hearts have been removed from images for presentation purposes. (E) Numbers of Mef2c-positive cells were counted with MIPAR. The 8 wpf data (red box) is shown on the right with a different scale for the *y*-axis. [8-week (blue) average wild type=1078, mutant=848; Welch's *t*-test, *P=0.0383, *n*=17; 3-month (green) average wild type=6061, mutant=4557; Welch's *t*-test, **P=0.0052, *n*=10 and 8, respectively). (F) Left: Western blot of whole-cell extracts from zebrafish hearts using an antibody directed towards canonical Eif4ea (top) and Tnnt2a (bottom). Right: Quantification of western blots with total Eif4ea levels normalized to the sarcomeric protein Tnnt2a as a measure of total cardiac mass (average increase=1.82; Welch's *t*-test, *P=0.0334, *n*=4). (G) Immunofluorescence of canonical Eif4ea/b (top, grayscale; bottom, green) and MHC (blue, bottom). Scale bar: 100 µm. For each panel, the lighter shade color is used for mutants, individual data points are represented by dots, and error bars represent mean±s.e.m. Mut, mutant; Wt, wild type.

*Eif4e1a* in mice is required for viability with no detectable homozygous mutant mice from heterozygous crosses (Sénéchal et al., 2021). To examine whether *eif4e1c* is essential in the zebrafish system, we generated a stable CRISPR mutant fish line with an *eif4e1c* deletion, hereafter referred to as Δ*eif4e1c*. Using guide RNA targeting flanking loci, we created a 12,131 bp deletion in the *eif4e1c* gene extending from intron 1 to exon 7 and deleting the entire coding region (Fig. 3A). Crosses between Δ*eif4e1c* heterozygotes yielded larvae at 3 days post-fertilization (dpf) in normal Mendelian ratios (Fig. S3A). Based on the gene expression analysis above (Fig. 2D) it is possible that embryonic Δ*eif4e1c* mutant fish develop through early stages owing to maternal deposition of wild-type *eif4e1c* mRNA or protein (Harvey et al., 2013). To address the possibility of maternal deposition effects, we crossed homozygous Δ*eif4e1c* mutant females with heterozygous Δ*eif4e1c* males and still found Mendelian ratios in the progeny, eliminating maternal deposition as a requirement for Δ*eif4e1c* mutant fish development (58 +/−:49 −/−). Mutants were found at normal Mendelian ratios through early juvenile stages up to 4 weeks post-fertilization (wpf) (χ^2^=0.629, *P*=0.73). Beginning in late juvenile stages, 8 wpf, heterozygotes and homozygous mutants were under-represented among the progeny (599 +/+:951 −/+:318 −/−; χ^2^=29.3, *P*=4.31×10^−7^). Although homozygous Δ*eif4e1c* fish were recovered in adults at 3 months post-fertilization (mpf), survivorship was diminished in mutants and heterozygotes (Fig. S3A; χ^2^=84.9, *P*=3.22×10^−19^). Therefore, deletion of *eif4e1c* is not embryonically lethal, but begins to influence viability between 4 and 8 wpf with only ∼80% of heterozygotes and ∼50% of homozygote mutants surviving past 8 wpf.

Throughout all stages of development, Δ*eif4e1c* mutants appear visibly normal morphologically but Δ*eif4e1c* mutant adults have notable size differences, suggesting impaired growth. At 3 mpf, Δ*eif4e1c* mutant fish were 12% shorter (Fig. 3B; mean length: wild type=1.816 cm, mutant=1.622 cm; *P*=0.0156; *n*=24 and 23, respectively) with 30% lower mass (Fig. 3C; mean weight: wild type=146.5 mg, mutant=103.1 mg; *P*=0.0014; *n*=24and 23, respectively). Similar results were found when males and females were considered separately and heterozygous Δ*eif4e1c* carriers did not demonstrate significant length or weight differences (Fig. S3B). At 8 wpf, Δ*eif4e1c* mutant fish had 36% less mass (Fig. 3C; mean weight: wild type=35.75 mg, mutant=22.82 mg; *P*=0.0392; *n*=24) and at 6 mpf Δ*eif4e1c* mutant fish had 24% less mass (Fig. 3C; mean weight: wild type=344.6 mg, mutant=261.7 mg; *P*=0.0002; *n*=20). Therefore, growth differences between Δ*eif4e1c* mutant fish and wild-type fish peak at juvenile stages and decrease into adulthood (Fig. S3C). Similarly, length differences found at 3 mpf were not detectable by 6 mpf (Fig. 3B; mean length: wild type=2.480 cm, mutant=2.370 cm; *P*=0.1212; Mann–Whitney; *n*=20). We conclude that Δ*eif4e1c* homozygous fish have reduced growth that persists but attenuates into adulthood.

To determine whether apparent growth defects reflect fewer overall cell numbers, we quantified CM numbers using an antibody for a nuclear marker of cardiac muscle (Mef2c). CM content was compared between adult hearts (3 mpf) of Δ*eif4e1c* mutant fish and their wild-type siblings. Mutant heart ventricles had 25% fewer CMs compared with their wild-type siblings at 3 mpf (Fig. 3E; mean: wild type=6061, mutant=4557; *P*=0.0052; *n*=10, 8, respectively). Fewer CMs were also measured at 8 wpf just after mutant death occurred (Fig. 3E, mean: wild type=1078, mutant=848; *P*=0.0388; *n*=17). At 6 mpf, mutant heart ventricles had fewer CMs, demonstrating that fewer CMs during development are not fully rescued by regeneration mechanisms in the adult (Fig. S3D). Reduced CM numbers may result from less CM proliferation, reduced CM survival or increased CM death. To determine whether hearts from Δ*eif4e1c* mutant fish were undergoing increased apoptosis, we performed terminal deoxynucleotidyl transferase dUTP nick end labeling (TUNEL) on heart sections from mutant and wild-type fish and observed no significant change (Fig. S3E,F). We conclude that deficits in overall growth of adult *eif4e1c* mutants likely reflect defects in either cell proliferation or cell survival during development. Phalloidin staining of sarcomeres revealed no gross differences between CM sarcomere structure in wild-type and mutant hearts (Fig. S3G). It is likely that proliferation and survival deficits are unrelated to structural differences in Δ*eif4e1c* mutant CMs.

In contrast to reported *Eif4e1a* mutants in mice, zebrafish *eif4e1c* deletion knockouts can survive (Altmann et al., 1989; Sénéchal et al., 2021). Similarities in sequence and expression patterns between *eif4e1c* and its canonical homologs *eif4ea* and *eif4eb* raises the possibility that Δ*eif4e1c* mutants survive because the canonical homologs can functionally substitute. To look at canonical protein levels, we used an antibody raised against the human canonical EIF4E1 that also recognizes the zebrafish orthologs (see Materials and Methods for details). Western blotting of whole-cell extracts from wild-type and Δ*eif4e1c* mutant hearts showed that canonical Eif4ea/b protein levels are increased in Δ*eif4e1c* mutants (Fig. 3F, fold change average=1.82, *n*=4). Interestingly, we observed an increase in Eif4ea/b protein levels at the site of injury during wild-type heart regeneration (Fig. 3G). In both wild-type and Δ*eif4e1c* mutant hearts, after amputation of the apex of the ventricle, Eif4ea/b protein levels increased to a similar extent at the site of injury (Fig. S3H). Taken together, canonical eIF4E1 protein levels increase in Δ*eif4e1c* mutant hearts, as they do during wild-type heart regeneration. We conclude that canonical Eif4ea/b likely partially compensates for cardiac growth deficits in surviving Δ*eif4e1c* mutant hearts.

Studies in other organisms have shown a wide array of lower expression levels of canonical eIF4E1A (50-70%) without obvious phenotypes in growth (Graff et al., 2007; Truitt et al., 2015). Therefore, we do not believe that reduced total levels of *eif4e1a* paralogs underlie growth deficits in Δ*eif4e1c* mutant adults. These observations lead us to hypothesize that the *eif4e1c* ortholog is specialized and functions to foster growth in juveniles and adults, a phenomenon commonly observed in aquatic species. From the reduced body size of Δ*eif4e1c* mutants, we speculate that loss of the Eif4e1c family in early terrestrial vertebrates may have limited growth in adults as an adaptation to survival on land.

### Ribosome profiling of Δ*eif4e1c* mutant hearts uncovers translational changes

**Fig. 4.**
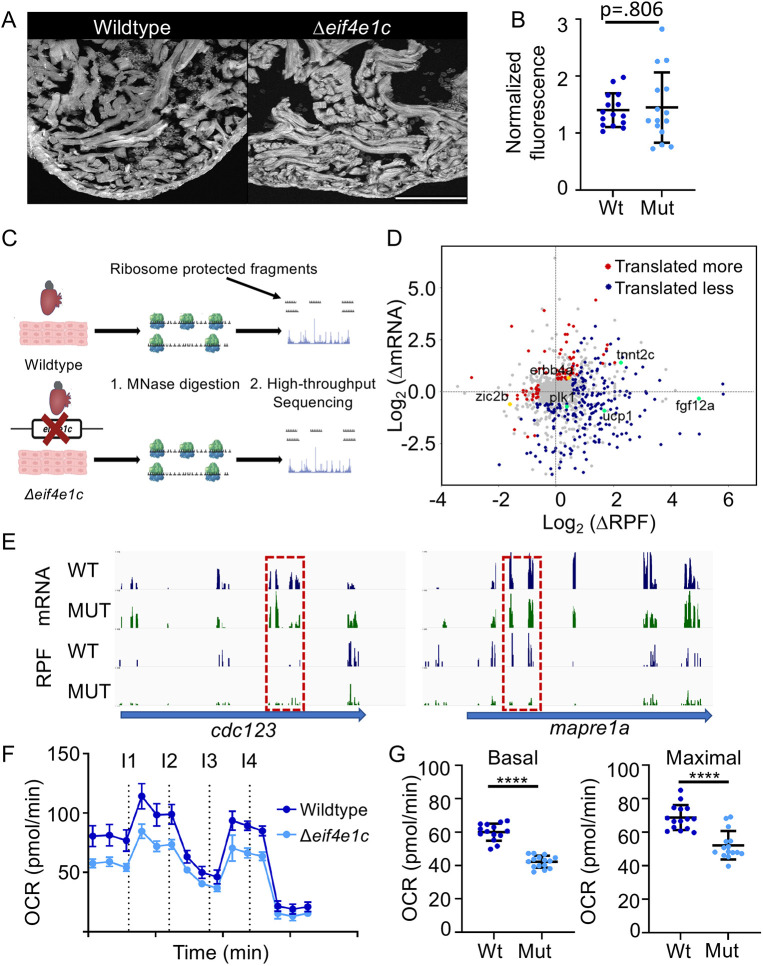
**Ribosome profiling indicates translational changes in *eif4e1c* mutant hearts.** (A) Example of fluorescence in the hearts of OPP-injected fish. Scale bar: 100 µm. (B) Quantification of OPP fluorescence shows no significant difference between mutant and wild-type fish (normalized fluorescence average wild type=140,279 arbitrary density units (adu)/µm^2^, mutant=145,096 adu/µm^2^; Mann–Whitney, *P*=0.806; *n*=15). Error bars represent mean±s.e.m. (C) Diagram of the ribosome profiling method. Ribosome-bound mRNA is purified from wild-type and mutant hearts and then subjected to micrococcal nuclease (MNase) digestion. Fragments of mRNA protected by being bound by ribosomes are then purified and subjected to high-throughput sequencing. (D) Differences (mutant/wild type) in mRNA abundance (log_2_) are plotted versus changes in the abundance of ribosome-protected fragments (RPF). Genes for which translation does not change significantly are plotted in gray, genes for which translation is decreasing in the mutant are plotted in red and genes for which translation is increasing in the mutant are plotted in blue. Decreasing genes mentioned in the text are colored gold and increasing genes mentioned in the text are colored green. (E) High-throughput sequencing browser tracks for two genes (blue arrows) that are involved in cell-cycle progression (*cdc123* and *mapre1a*). Wild-type (WT) data is shown in blue, and mutant (MUT) data is shown in green. The top two tracks (mRNA) show the abundance of mRNA (as measured from RNAseq) and the bottom two tracks show the abundance of RPFs. Dashed red boxes highlight decreasing levels of RPF where mRNA levels are comparable. (F) Time course of oxygen consumption rates (OCR) from mitochondria measured by the Seahorse analyzer. Dotted lines indicate time points of drug injection. Readings were normalized to the number of mitochondria using qPCR. The first injection (I1) is of ADP to stimulate respiration, the second injection (I2) is of oligomycin to inhibit ATP synthase (complex V) decreasing electron flow through transport chain, the third injection (I3) is of FCCP to uncouple the proton gradient, and the fourth injection (I4) is of antimycin A to inhibit complex III to shut down mitochondrial respiration. Error bars represent mean±s.e.m. (G) Basal respiration is calculated as the OCR average after addition of ADP (I1 to I2) subtracting the non-mitochondrial respiration after injection of antimycin A (after I4) (mean wild type=60.01, mutant=42.25; Welch's *t*-test, ****P<0.0001; *n*=15). Maximal respiration is calculated as the OCR average after FCCP addition (I3 to I4) subtracting the non-mitochondrial respiration after injection of antimycin A (after I4) (mean wild type=68.73, mutant=52.19; Welch's *t*-test, ****P<0.0001; *n*=15). Error bars represent mean±s.e.m. Mut, mutant; Wt, wild type.

The eIF4e1 family core amino acids responsible for initiating loading of ribosomes for translation are conserved in zebrafish Eif4e1c. Therefore, we hypothesized that the survival and growth deficits that occur upon deletion of *eif4e1c* result from alterations in translation. To measure global translation in *eif4e1c* mutants, we injected fish with O-propargyl-puromycin (OPP), which terminates peptide chain elongation. Total translation (incorporation of OPP) can be measured from fluorescence levels by conjugating fluorophores using click chemistry (Fig. 4A). Injection of OPP into Δ*eif4e1c* mutants and wild-type siblings demonstrated that global protein synthesis is unperturbed in Δ*eif4e1c* mutants (Fig. 4B; mean fluorescence: wild type=140,279 arbitrary density units (adu)/µm^2^, mutant=140,596 adu/µm^2^; *P*=0.806, Mann–Whitney; *n*=15). We conclude that growth deficits in Δ*eif4e1c* mutants are not a result of impaired general translation.

To determine whether particular transcripts are translated differently in *eif4e1c* mutants, we performed ribosome profiling, which involves sequencing mRNA fragments protected by virtue of being bound to the ribosome (Fig. 4C). Therefore, the level of ribosome-protected fragments (RPFs) can illuminate the translational status of a transcript. Normalization of RPFs to the total amount of transcript (RNAseq) provides a quantitative measure of translational efficiency that can be compared between conditions. Because Δ*eif4e1c* mutants have small hearts as adults, we performed ribosome profiling to compare the translatome of adult 3 mpf Δ*eif4e1c* mutant hearts with that of their wild-type siblings. Overall, there were 88 genes with transcripts that were less efficiently translated and 224 genes with transcripts that were more efficiently translated in the Δ*eif4e1c* mutant (Fig. 4D). Thus, deletion of the *eif4e1c* gene resulted in translational changes in about one-fifth (22.6%) of the total expressed genes detected in ribosome profiling of adult hearts.

Several of the translationally misregulated transcripts in Δ*eif4e1c* mutant hearts could explain some of the observed phenotypes. For example, RPFs for *erbb4*, encoding a receptor for the potent CM mitogen Nrg1, are 44% lower in Δ*eif4e1c* mutant hearts (Bersell et al., 2009; D'Uva et al., 2015; Gemberling et al., 2015). Retinoic acid signaling is also required for CM proliferation during development (Keegan et al., 2005) and regeneration (Kikuchi et al., 2011) The translational efficiency for a transcription factor downstream of retinoic acid signaling called *zic2b* is 81.6% lower in mutant hearts (Fig. 4D). In addition, several cell cycle genes had transcripts that were not translated as well in Δ*eif4e1c* mutant hearts (Fig. 4E). For example, translated less were *mapre1a*, which associates with AuroraB kinase to assemble microtubules during cytokinesis (Sun et al., 2008), and *cdc123*, which encodes a factor needed for protein synthesis initiation and cell cycle progression (Okuda and Kimura, 1996; Perzlmaier et al., 2013). We cannot formally determine from which cell types these translational changes are occurring. Yet, we predict that at least some changes are occurring in CMs because CMs comprise most cells in uninjured zebrafish hearts and decreased translation of cell proliferation transcripts, such as *mapre1a* and *cdc123*, are likely occurring there. In sum, deletion of *eif4e1c* led to decreased translation of mRNA encoding signaling pathways that promote CM proliferation and cell division in uninjured hearts.

Other *eif4e1c*-dependent changes in translation may occur at time points closer to 4 wpf when mutants first begin to die. Ribosome profiling at these stages is not possible because zebrafish hearts are too small to yield the amount of starting material required. By 12 wpf, translational buffering (Kusnadi et al., 2021; Lorent et al., 2019) or compensation from canonical cap-binding proteins (Fig. 3F) may mask some of the transcripts affected by *eif4e1c* to support the 50% mutant survival beyond 4 wpf. Evidence for compensation is present in the ribosome profiling data set. For example, we detected increased translational efficiency of transcripts encoding pro-growth factors in Δ*eif4e1c* mutant hearts, such as Fgf family members (*fgf8b:* 2.96-fold; *fgf12a:* 31.59-fold; *fgf19:* 2.38-fold), which have been reported to be pro-survival and pro-proliferative to CMs (Lepilina et al., 2006; Sakurai et al., 2013; Tahara et al., 2021). In addition, reduced mitochondrial activity has been reported to be pro-proliferative in the heart (Cardoso et al., 2020; Fukuda et al., 2020; Honkoop et al., 2018; Miklas et al., 2021). Translated more efficiently are transcripts for *ucp1* (3.23-fold), encoding a mitochondrial uncoupling factor that reduces mitochondrial activity to mitigate production of reactive oxygen species (Echtay et al., 2002). In conclusion, translation efficiency of different pro-growth pathways and molecules is both enhanced and diminished in Δ*eif4e1c* mutant hearts. Given that mutant hearts are still deficient in growth (Fig. 3D,E), we postulate that *eif4e1c* is essential for heart growth, but that other less efficient pro-growth pathways can partially compensate for *eif4e1c* loss through the canonical *eif4e1* orthologs.

To confirm the biological significance of increased *ucp1* translation, we measured oxygen consumption rates (OCRs) of mitochondria isolated from hearts using the Agilent Seahorse bioanalyzer (Fig. S4B). Mitochondria from Δ*eif4e1c* mutant hearts had lower OCRs compared with the mitochondria from wild-type sibling hearts (Fig. 4F). The basal and maximal respiration rates of mutant hearts were significantly lower (by 25.59% and 24.06%, respectively), which suggests impaired mitochondrial output (Fig. 4G). To examine whether reduced mitochondrial activity is also detected using Seahorse during wild-type heart growth, we compared respiration of mitochondria from uninjured hearts to mitochondria from regenerating hearts using the Seahorse analyzer. In agreement with a previous report (Honkoop et al., 2018), mitochondrial activity plummets in zebrafish hearts undergoing regeneration with basal respiration decreasing by 75.5% and maximal respiration decreasing by 62.0% (Fig. S4C-E). Given that reduced mitochondrial activity correlates with growing heart muscle, a similar, but less profound, reduction in mitochondrial activity in Δ*eif4e1c* mutants may function as a mechanism to foster compensatory growth.

Ribosome profiling demonstrated that translational changes occur in Δ*eif4e1c* mutant hearts, but it is unlikely every transcript is a direct Eif4e1c target. Second-order effects are possible, especially if compensation drives survival in Δ*eif4e1c* mutants. Therefore, it is likely some Eif4e1c-bound transcripts are not affected in our ribosome profiling experiment. Nevertheless, many translational changes are detected in hearts of Δ*eif4e1c* mutant hearts, including some that may explain their smaller heart size.

### Deletion of *eif4e1c* impairs metabolic remodeling and regeneration in the heart

**Fig. 5.**
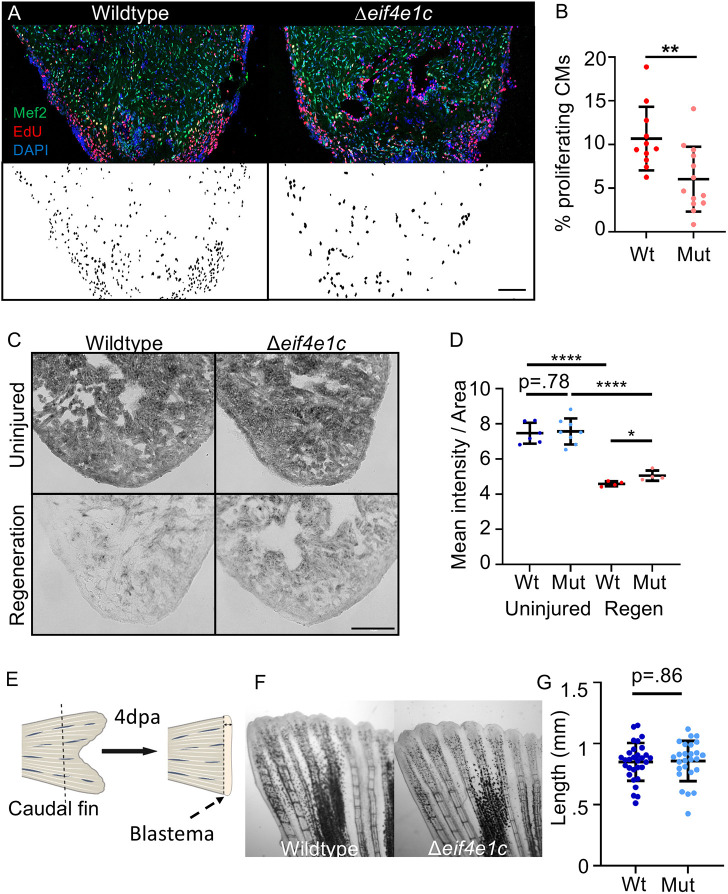
**Deletion of *eif4e1c* impairs CM proliferation in regenerating hearts.** (A) Images of sectioned amputated ventricles (7 dpa) from wild-type and Δ*eif4e1c* mutant fish. Sections are stained for Mef2c (green) and EdU (red). Double-positive cells are highlighted in black below using a MIPAR software rendition. Scale bar: 100 µm. (B) Quantification of CM proliferation indices (Mef2/EdU double-positive over total Mef2 positive) in 7 dpa ventricles (average wild type=10.68%, mutant 6.02%; Welch's *t*-test, **P=0.0054; *n*=11 and 13, respectively). Error bars represent mean±s.e.m. (C) Cryosections of fresh mutant and wild-type hearts were stained for Succinate dehydrogenase activity. Shown are uninjured hearts (top) and hearts that were ablated genetically using Z-CAT (7 days-post-incubation) (bottom). Scale bar: 20 µm. (D) The mean signal intensity was calculated per given area for Sdh-stained hearts (uninjured average wild type=7.47, mutant=7.57; *n*=6 and 8, respectively; regeneration average wild type=4.59, mutant=5.06; *n*=4). Welch's *t*-test was used to calculate significance (wild type versus mutant uninjured=0.781, wild type uninjured versus wild type regeneration ****P<0.0001, mutant uninjured versus mutant regeneration ****P<0.0001). For every replicate (*n*=4) the mutant Sdh activity during regeneration (pink) was higher than the wild-type activity during regeneration (red). Error bars represent mean±s.e.m. (E) Diagram of caudal fin regeneration experiments. The blastema is the highly proliferative zone of growth that forms the new fin. (F) Caudal fins were amputated ∼50% in Δ*eif4e1c* mutants and their wild-type siblings and shown are images of fin regrowth 4 days later. (G) There was no significant difference between fin growth in wild type (blue) and mutant (light blue) fish (average wild type=0.849 mm, mutant 0.857 mm; Welch's *t*-test, *P*=0.861; *n*=30 and 26, respectively). Error bars represent mean±s.e.m. Mut, mutant; Wt, wild type.

We hypothesized that increased expression of *eif4e1c* mRNA in regenerating hearts may stimulate heart growth during regeneration (Goldman et al., 2017). After surgical removal of the apex of the heart, Δ*eif4e1c* mutants had diminished CM proliferation during the peak of heart regeneration compared with wild-type siblings (Fig. 5A). The fraction of Mef2c-positive CMs that were also positive for the proliferation marker 5-ethynyl-2′-deoxyuridine (EdU) was reduced by 35% 7 days post-amputation (dpa) of the ventricular apex (Fig. 5B, mean: wild type=10.68%, mutant=6.021%; *P*=0.0054, *n*=11 and 13, respectively). We cannot determine whether *eif4e1c* proliferation phenotypes are CM specific or a result of secondary effects from other cell types. Homozygous Δ*eif4e1c* mutants did complete regeneration, despite early defects in CM proliferation, and look the same as their wild-type siblings by 28 dpa (Fig. S5A). Thus, we conclude that Δ*eif4e1c* mutants have impaired CM proliferation that may be resolved by compensatory mechanisms at later stages of regeneration.

As previously discussed, decreased mitochondrial respiration is a feature of heart regeneration that promotes growth. One of the key enzymes central to connecting the tricarboxylic acid cycle to the electron transport chain is succinate dehydrogenase (Sdh); Sdh activity is reported to be sharply downregulated during heart regeneration (Bezawork-Geleta et al., 2017; Honkoop et al., 2018). Using the zebrafish cardiomyocyte ablation transgenes (Z-CAT) system (Wang et al., 2011), we injured CMs throughout the heart and confirmed that Sdh activity is sharply reduced during regeneration (Fig. 5C). Although Δ*eif4e1c* mutant hearts also have lower Sdh activity during regeneration, Sdh activity was higher in Δ*eif4e1c* mutant hearts compared with their wild-type siblings (Fig. 5D). For uninjured hearts, Sdh activity was unchanged between mutant and wild type. This suggests that an inability to fully reduce Sdh activity may in part underlie the impairment of heart regeneration in Δ*eif4e1c* mutants. It is unclear whether the *sdh* transcript is a direct target of *eif4e1c* or if changes in its activity are regulated indirectly.

After development, Δ*eif4e1c* mutants had deficiencies in overall size (Fig. 3B,C). To determine whether impaired growth is a general feature of regeneration, we analyzed the ability of Δ*eif4e1c* mutants to regenerate their fins. Fin regeneration was determined by measuring growth of caudal fins in Δ*eif4e1c* mutants and their wild-type siblings at 4 dpa (Fig. 5E,F). There was no significant difference in fin regrowth, indicating that Δ*eif4e1c* mutants can normally regenerate their fins (Fig. 5G; mean wild type=0.849 mm, mutant=0.857 mm; *P*=0.861, *n*=30 and 26, respectively). An earlier time point of fin regeneration (2 dpa) similarly showed no difference between wild-type and Δ*eif4e1c* mutant siblings (Fig. S5B; mean: wild type=0.689 mm, mutant=0.617 mm; *P*=0.366, *n*=18 and 22, respectively). We performed a survey of published RNAseq data sets (see Materials and Methods) from zebrafish fins and found that although overall *eif4e1c* expression is highest in the uninjured fin (Fig. 2C), transcripts for *eif4e1c* are not increased during fin regeneration like they are in the heart (Fig. S5C,D; mean uninjured wild-type fin=0.795, mean regenerating wild-type fin=0.935, mean uninjured wild-type heart=0.308, mean regenerating wild-type heart=0.865). We conclude that although Δ*eif4e1c* mutants may have general growth deficits during development (Fig. 3B,C), during regeneration *eif4e1c* function is more crucial to growth in the heart.

In uninjured hearts and fins, transcript levels for *eif4e1c* were reduced by 96% and 97% from wild-type levels in the mutants (Fig. S5E). As expected, the mutant transcript retains the 3′UTR but is missing most of the coding sequence and is therefore likely degraded. Interestingly, *eif4e1c* transcript levels during heart regeneration increased 10.7-fold in mutants, which is nearly 5-fold higher than *eif4e1c* increased in the wild-type hearts during regeneration (Fig. S5F; mean: wild type=2.30, mutant=10.70; *n*=10). This suggests a mechanism of compensation whereby a feedback loop stimulates *eif4e1c* transcription when Eif4e1c activity is absent. Such a feedback loop does not exist in regenerating fins as there was no difference in the change of *eif4e1c* transcript abundance between wild type and mutants (Fig. S5F; mean: wild type=0.667, mutant=0.787; *n*=10). The existence of a feedback loop in the heart and not in the fin further argues that Eif4e1c activity is more important to the heart during regeneration. We cannot exclude the possibility that other compensation mechanisms may mask the presence of this feedback loop in fins.

This study establishes a new pathway for translational regulation mediated by a homolog of an mRNA cap-binding protein called *eif4e1c*. This pathway is highly conserved in aquatic species but lost in terrestrial vertebrates. Importantly, we show that, in zebrafish, *eif4e1c* is required for normal cardiogenesis during development and regeneration. Although *eif4e1c* is broadly expressed both during development and in adulthood, overt mutant phenotypes are restricted to particular tissues and first observed in juveniles. For example, heart regeneration, but not fin regeneration, is impaired. It is likely that specific features of the Eif4e1c protein allow for targeting distinct cohorts of transcripts that underlie the different tissue sensitivities found during regeneration. The data presented here suggest that regulation of metabolism may be an aspect of *eif4e1c* function that predisposes heart muscle to a reliance of *eif4e1c*. Alternative pro-CM growth pathways not dependent on *eif4e1c* exist, as Δ*eif4e1c* mutants do complete regeneration, albeit with a reduced index of CM proliferation. In the future, determining a contribution to regeneration for each of the canonical mRNA cap-binding proteins will help construct a translation regulatory network for heart regeneration with possible therapeutic implications.

## MATERIALS AND METHODS

### Phylogeny of the Eif4e1c family

Expanding on previous studies, we analyzed 20 species of teleosts covering most, if not all, subclades (Lin et al., 2017), including the most ancestral species Asian arowana (*Scleropages formosus*) (Bian et al., 2016) and the spotted gar (*Lepisosteus oculatus*) (Braasch et al., 2016). Beyond teleost, we also included multiple species of sharks, rays, eels, and the fish that are the closest relatives to terrestrial tetrapods, such as mudskippers (*Periophthalmus magnuspinnatus*) (You et al., 2014) and lungfish (*Polypterus senegalus*, *Protopterus annectens*) (Austin et al., 2015). Homologs for *eif4e1c* were not found in more primitive deuterostomes, for example the sea squirt *Ciona intestinalis*, suggesting that *eif4e1c* may have arisen in the tetrapod genome duplication that predates the teleost-specific event. Phylogenetic analysis was conducted using maximum likelihood as an optimality criterion as implemented in IQ-TREE2 (Minh et al., 2020). The JTT+G (four-state) model of amino acid substitution was selected using corrected Akaike Information Criterion (AIC) scores and used to estimate the phylogeny. Branch support was estimated using 1000 replicates of the ultrafast bootstrap with a minimum correlation coefficient of 0.99. IQ-TREE search parameters include a perturbation strength of 0.5 for the randomized nearest neighbor interchange with >100 unsuccessful iterations required to end the heuristic search of tree space. All analyses were conducted using the IQ-TREE web server (Trifinopoulos et al., 2016).

### Gene expression analysis from RNA sequencing

To examine *eif4e1c*, *eif4ea* and *eif4eb* expression in adult zebrafish tissues, we turned to previously published RNA sequencing datasets available on NCBI Gene Expression Omnibus. Briefly, fragments per kilobase of transcript per million mapped reads (FPKM) was calculated from the raw read counts and normalized to the FPKM of *mob4*, a gene that does not change expression between tissues (Hu et al., 2016). For zebrafish fins, we obtained data from three different studies [two replicates from GSE126701 (Lee et al., 2020), two replicates from GSE146960: GSM4411406 and GSM4411407 (Thompson et al., 2020), and one replicate from GSE76564 (Kang et al., 2016)]. For zebrafish heart, we obtained datasets from two different studies [two replicates from GSE75894 (Kang et al., 2016) and two replicates from GSE168371: GSM5137453 and GSM5137454 (Pronobis et al., 2021)]. For zebrafish brain, we looked at datasets from two different studies [two replicates from GSE158079: GSM4790340, GSM4790341 (Sun et al., 2022) and two replicates from GSE151307 (Saraswathy et al., 2022)]. For zebrafish kidney, we obtained datasets from two different studies [two replicates from GSE158079: GSM4790350 and GSM4790351, and three replicates from GSE193630: GSM5815254, GSM5815255, GSM5815256 (Sun et al., 2022)]. The zebrafish liver datasets were derived from one study, GSE183023 (Heinkele et al., 2021) consisting of six replicates (GSM5549094). The spinal cord datasets were obtained from two studies: GSE183644 (two replicates) (Saraswathy et al., 2022) and GSE77025 (one replicate) (Mokalled et al., 2016). Only two replicates were available for testis, ovary and oocyte from GSE111882 (Herberg et al., 2018).

### scRNAseq analysis

For scRNAseq analysis of uninjured and injured hearts, we obtained count files from GSE159032 and GSE158919 (Hu et al., 2020) and reanalyzed with Seurat package (Stuart et al., 2019). Low-quality cells (nFeature_RNA≥4100, mitoRatio≥0.25) were filtered out. The 50 principal components (PCs) of the principal components analysis with resolution 4 were used for clustering.

### Statistics

To ensure that experiments were sufficiently powered we used an α error probability of 0.05 and 80% power to calculate if sample sizes were sufficient. For each analysis, we used the Welch's parametric *t*-test to calculate significance. Outliers were removed using the ROUT (Robust regression and outlier removal) method available in GraphPad Prism software. To define outliers, Prism uses the false discovery rate approach to handling multiple comparisons (Q=1%) to remove the outliers, and then analyzes the data using ordinary least-squares regression. If the variance between the comparison groups was also significant (*F* test for unequal variances), we switched to using a non-parametric test (Mann–Whitney). For the CM counting and CM proliferation assays, one researcher embedded hearts and sectioned slides and a separate researcher who was unaware of the sample identity carried out imaging and quantification.

### Fish strains

All zebrafish (*Danio rerio*) used in this study derive from the Ekwill strain. Adults less than 1 year old were used for all the experiments with specific ages noted for the relevant experiments. Males and females were mixed in similar proportions in each of the conditions. All experiments were performed under university supervision according to the institutional animal care and use committee protocol #2018R00000090 of Ohio State University.

### Construction of *eif4e1c* deletion fish line

Guide RNAs (sgRNAs) were designed against intron 1 (GGACATGTTTAAGCAGTGAG) and exon 7 (CAGAGGCACCTGCAGACTGA). DNA templates of the respective sgRNA fused to a tracRNA were produced by PCR. T7-transcribed sgRNA were then injected with Cas9 protein into newly fertilized embryos from EK parents. Adults injected at fertilization were mated and embryos of possible stables lines were screened by PCR oligos (Fwd: CGGTAAGAGGGATCGGGCTTGGT, Rev: GTCTACAAGCAAGATGCCACAATTCCAGCA) flanking the deleted region (chr13:18,311,769:18,323,899). The selected line, called *eif4e1c^pd370^*, was a perfect deletion without addition of extra nucleotides between the fused cut sites. Heterozygote adults were mated as described.

### Length and weight measurements

Zebrafish (3-6 mpf) were anesthetized in phenoxyethanol and their length was measured using a ruler starting from the eye to the center of caudal tail fin before the lobes diverge. After removing excess water with a Kimwipe, the anaesthetized zebrafish were weighed on an analytical scale. Fish of different genotypes were raised and housed together and measured shortly after genotyping.

### Immunohistochemistry

Primary antibodies used in this study were: anti-Mef2 (1:100, Abcam, ab197070), anti-hsEIF4E (1:100, Abcam, ab 33768), and anti-MF20 (1:100, Developmental Studies Hybridoma Bank, MF20). Secondary antibodies were: anti-mouse Alexa Fluor 546 (Thermo Fisher Scientific, A-11030; 1:200) for MF20, and anti-rabbit Alexa Fluor 488 (Thermo Fisher Scientific, A-11034; 1:200) for Mef2. Three sections representing the hearts with largest lumen were selected from each heart and imaged using a 20× objective on a Zeiss LSM 900 with Airyscan confocal microscope. Files were imported into ImageJ, channels split (DAPI, GFP and RFP), and the brightness and contrast of each channel adjusted consistently between wild-type and mutant hearts. RFP and GFP channels (excluding DAPI) were merged for wild type and mutant. Images were rotated such that the heart apices were pointed to the bottom and the other hearts that were partially in the image were edited out. Images were cropped to the same extent for wild-type and mutant hearts so that the image dimensions are the same.

For obtaining cardiomyocyte counts, the number of Mef2c^+^ nuclei were counted using MIPAR image analysis software (REF Sosa). To calculate differences in immunofluorescence for anti-hsEIF4E, we drew regions of interest of the same size using ImageJ. The ratio of raw intensity (RawIntDen) was determined at the injury site versus an area distal to the injury after normalization to background fluorescence. Phalloidin staining was performed according to the manufacturer's protocol using 1:200 dilution (Abcam, 176753). TUNEL assays were also performed according to the manufacturer's protocol (Thermo Fisher Scientific, C10617).

### Western blot

Originally raised against the human form of canonical EIF4E, we used an antibody (Abcam, ab33768) that targets a region highly homologous to both zebrafish canonical paralogs, Eif4ea and Eif4eb (95% similar and 82% identical; Fig. S6A). The regions in common are shared by all three proteins so the antibody should recognize both zebrafish paralogs. The antibody detects a major band of the expected size (∼25 kD) by western blotting when probed against whole-cell extract from zebrafish hearts. In addition, the antibody demonstrated cytoplasmic staining patterns on zebrafish hearts that are consistent with what was found when used for immunofluorescence on human cells (Fig. S6B).

### OPP experiments

OPP (Cayman Chemical) was resuspended in DMSO and then diluted with PBS to working solution of 5 µM. Each fish was injected intraperitonially with 10 µl and allowed to swim around for 1 h before heart removal. Longer incubation times did not affect the data. Hearts were fixed, sectioned and then stained using click chemistry (Breinbauer and Köhn, 2003) with Alexa Fluor 594 Azide (Thermo Fisher Scientific, A10270). To calculate fluorescence, images were opened in ImageJ and thresholding was used to calculate fluorescence. The average mean intensity was used for background readings to correct for total fluorescence. Three sections were averaged as technical replicates from each heart.

### Ribosome profiling

For each replicate sample, RNA from 30 zebrafish hearts was extracted for Ribo-seq library generation and parallel RNA sequencing. The hearts were lysed on ice with 100 μl of lysis buffer with 53 mg/ml cycloheximide using mechanical homogenization with a pestle for 30 s. Cells were lysed further by trituration of the sample using a 27.5-gauge needle syringe 20 times on ice. Cell extracts (average OD 7) were treated with micrococcal nuclease (MNase; NEB), CaCl_2_ (5 mM final concentration), and Turbo DNAse I (Invitrogen) at 25°C for 30 min. MNase digestion was stopped with SUPERase-inhibitor incubation on ice. 80S ribosomes were isolated using SH400 spin columns (Cytiva). Columns were prepared according to the manufacturer's instructions. One-hundred microliters of MNase-treated sample was loaded per column and centrifuged for 2 min at 600 ***g***. The flow through was isolated and incubated for 5 min with 3× volume of Trizol LS (Ambion), then 200 µl of chloroform was added and incubated for 2 min. Samples were centrifuged for 15 min at 12,000 rpm (13,400 ***g***) at 4°C. The aqueous phase was extracted and precipitated overnight at −20°C using GlycoBlue (Thermo Fisher Scientific) and isopropanol followed by centrifugation for 30 min at 4°C at 10,000 rpm (9400 ***g***). Pellets were washed with 75% ethanol and resuspended into RNase-free water (Ingolia et al., 2012).

Samples were subjected to 15% denaturing polyacrylamide-mediated gel (UreaGel 29:1 gel system, National Diagnostics) and visualized with SYBR Gold (Thermo Fisher Scientific) after electrophoresis. Ribosome-protected RNA fragments 26-mers to 34-mers were excised and RNA was eluted overnight by rotating the gel slices in RNA extraction buffer [300 mM sodium acetate (pH 5.2), 1 mM EDTA, 0.25% (w/v) SDS] at room temperature. To deplete rRNA fragments, the isolated RNA was precipitated on dry ice for 30 min using GlycoBlue (Thermo Fisher Scientific) and isopropanol and treated with the RiboMinus Ribosomal RNA depletion kit (Ambion), according to the manufacturer's instructions.

The RNA samples were then de-phosphorylated by adding 1 μl of T4 PNK, 1 μl SUPERase•In, 7 μl of 10× PNK reaction buffer for a 70 μl volume reaction. The reaction was incubated then for 1 h at 37°C before adding 1 μl of 100 mM ATP for 30 min at 37°C. The reaction was then heat inactivated at 70°C for 10 min and precipitated as before. Adapter ligation, reverse transcription and subsequent cDNA amplification were performed using the NEXTFLEX Small RNA-Seq Kit v3 (Perkin Elmer). Samples were subjected to 8% native PAGE (0.5× TBE) gel electrophoresis. cDNA products of the final PCR were visualized with SYBR Gold (Thermo Fisher Scientific), excised from the gels, and extracted overnight with DNA extraction buffer [300 mM NaCl, 10 mM Tris (pH 8.0), 1 mM EDTA] and precipitated as described previously.

To prepare RNAseq libraries, total RNA was isolated from each sample using Trizol LS as described above and subjected to TURBO DNase I digestion (1 μl TURBO DNase I, 1× DNase reaction buffer, 20 min at 37°C). RNAseq libraries were constructed from 500 ng of purified RNA using Illumina's TruSeq Stranded Total RNA kit, according to the manufacturer's instructions, by the Case Western Reserve University (CWRU) Genomics Core.

Libraries were assessed for quality by Agilent Bioanalyzer followed subsequently by single-end 75 cycle sequencing. RNAseq and Ribo-seq sequencing were performed at the CWRU Genomics Core on the Illumina NextSeq 550 v2.5 (High Output) platform.

### Bioinformatics

Ribosome profiling FASTQ files were processed using FastQC (v0.11.8; RRID:SCR_014583) for quality control analysis. Pre-processing of sequencing reads was based on the quality control report using the FASTQ Quality Filter module in the FASTX-Toolkit (RRID:SCR_019035). This was used to filter the bases with 99% accuracy based on Q Score. Reads were removed that showed less than 70% of nucleotides with an accuracy of at least 99%. The sequences were collapsed according to their unique molecular identifiers (UMIs). Cutadapt (RRID:SCR_011841) was used to trim 21-nt adaptor before the first nucleotide and the last four nucleotides from the reads to remove the UMI.

The processed reads were aligned to GRCz11/danRer11 (assembly of the zebrafish genome; Genome Reference Consortium) using STAR v2.5.3a (RRID:SCR_004463). Sequencing replicates were highly similar to one another for both ribosome-protected fragments and input mRNA (Fig. S5A). Generated bam files were processed with RiboProfiling package v1.2.2 (Popa et al., 2016). Coverage counts on the coding regions (CDS) were obtained for each sample based on RiboProfiling function modules, *TxDb.Drerio.UCSC.danRer11.refGene* (v3.4.6; Annotation package for TxDb object(s); https://bioconductor.org/packages/release/data/annotation/html/TxDb.Drerio.UCSC.danRer11.refGene.html) and GenomicFeatures package v1.46.1 (Lawrence et al., 2013). Differential expression analysis was performed using the DESeq2 pipeline (version 1.26.0), based on the expressed raw reads (Love et al., 2014). Statistic thresholds for the features in the comparison results (*P*-values and log2 fold changes), were used for the gene extraction, as described.

Genes with transcripts with increasing translation efficiency in mutant hearts were calculated as (1) significantly increasing by RPF and not mRNA, (2) not significantly increasing by RPF but significantly decreasing by mRNA or (3) significantly changing in both datasets with an RPF/mRNA ratio >1. Genes with transcripts translated less efficiently were identified as (1) decreasing significantly by RPF and not changing by mRNA, (2) not significantly changing in RPF but significantly increasing in mRNA, or (3) significantly changing in both datasets with an RPF/mRNA ratio <1. Gene ontology categories were identified using DAVID (Huang et al., 2009; Sherman et al., 2022).

### Zebrafish cardiac regeneration experiments

Zebrafish were anesthetized using Tricaine and placed ventral side up on a sponge to carry out resection of the ventricular apex. Iridectomy scissors were used to make an incision through the skin and pericardial sac. Gentle abdominal pressure exposed the heart and ∼20% of the apex was removed with scissors, penetrating the chamber lumen (Poss et al., 2002). Hearts were harvested 7, 14 or 30 days after injury depending on the experiment. To genetically ablate CMs, *cmlc2:CreER^pd10^; bactin2:loxp-mCherry-STOP-loxp-DTA^pd36^* (Z-CAT) fish were incubated in 0.5 μM tamoxifen for 17 h (Wang et al., 2011).

#### To quantify CM proliferation

Injured fish were injected into the abdominal cavity once every 24 h for 3 days (4-6 dpa) with 10 μl of a 10 mM solution of EdU diluted in PBS. Hearts were removed on day 7, embedded and cryosectioned. Slides were stained with Alexa Fluor 594 Azide using click chemistry (Breinbauer and Köhn, 2003) and then immunostained for Mef2c. Briefly, sections were blocked with 1% bovine serum albumin (Fraction V) and 5% goat serum and washed in PBS with 0.2% Triton X-100. Three sections representing the largest wound area were selected from each heart and imaged using a 20× objective. The number of Mef2^+^ and Mef2^+^EdU^+^ cells were counted using MIPAR image analysis software and the CM proliferation index was calculated as the number of Mef2^+^EdU^+^ cells/total Mef2^+^ cells (Sosa et al., 2014). The CM proliferation index was averaged across two to four appropriate sections from each heart.

#### To visualize regrowth

Hearts were removed 28 dpa, embedded and cryosectioned. Slides were incubated at 60°C for 3 h in Bouin fixative previously heated at 60°C for 30 min. Slides were then rinsed in distilled water (dH_2_O) for 30 min, and then incubated with 1% (w/v) phosphomolybdic acid solution for 5 min. Following a 5 min wash step in dH_2_O, sections were stained with the Acid Fuchsin Orange G solution for 5 min, washed, dehydrated and then mounted in Cytoseal. Three sections representing the largest wound area were selected from each heart and imaged using a 20× objective.

### Fin regeneration analysis

Fish were anesthetized in phenoxyethanol, and the caudal fins were amputated just below bifurcations of rays using a razor blade and removing approximately half of the fin from body to tip. Animals were allowed to regenerate for 4 days, after which fins were imaged using a 10× objective. Fin regrowth was measured from the amputation till the tip of each ray using Zeiss microscope software. The second to fourth rays were averaged from both the dorsal and ventral sides.

### Seahorse analysis of mitochondrial respiration

Mitochondria from fish hearts were isolated as previously described in mice (Peri-Okonny et al., 2019). Oxygen consumption rates were optimized for 1.5 µg of mitochondria, which were resuspended in respiratory buffer (70 mM sucrose, 220 mM mannitol, 10 mM K_2_HPO_4_, 5 mM MgCl_2_, 2 mM HEPES, 1 mM EGTA, pH 7.4). Electron coupling (EC) assays were performed using the Seahorse Bioanalyzer (Agilent). The EC assay measures basal respiration in coupled mitochondria with substrates present (succinate/rotenone), but no ADP, which drives respiration through electron transport chain (ETC) complexes II-IV. Active respiration is initiated by addition of ADP to mitochondria, increasing OCR. Oligomycin, an ATP synthase/Complex V inhibitor, decreases OCR. FCCP, an ETC chemical uncoupler, allows for maximal uncoupled respiration. Antimycin A inhibits complex III, and shuts down electron flow, ATP production and OCR. Proton leak through the ETC can be determined with oligomycin treatment, and spare respiratory capacity is the difference in maximal respiration (FCCP treatment) and protein leak (oligomycin treatment). After Seahorse measurements, the abundance of mitochondria was verified using qPCR and used to normalize the readings.

### Sdh staining

Hearts were embedded fresh and sectioned and stained the same day. Succinate dehydrogenase (Sdh) enzymatic activity was measured by incubating sections with 37.5 mM sodium phosphate buffer pH 7.60, 70 mM sodium succinate, 5 mM sodium azide and 0.4 mM Tetranitro Blue Tetrazolium for 20 min at 28°C (Honkoop et al., 2018). The reaction was quenched in 10 mM HCl, and slides were mounted in glycerin-gelatin mounting media. Images were processed with ImageJ calculating percentage mean intensity of signal averaged by the total area of the heart (pixels).

### Gene expression analysis by RT-qPCR

Dissected tissues were placed in Hank's Buffered Salt Solution and washed three times in PBS before being snap-frozen. For regenerating fins, only the blastema was removed. Organs from ten fish were pooled (five male, five female). Primer pair efficiency was assessed and validated to be 95-105%. RNA was isolated using an adapted version of a published protocol and then treated with DNase. Reverse transcription (RT) was performed with 1 μg total RNA using SuperScript III (Thermo Fisher Scientific) and qPCR was performed with three technical replicates for each biological replicate. Controls without RNA template in the RT were run for each primer pair to identify possible DNA contamination and none was found. Fold gene expression was determined using the ΔΔCt method and normalized to *mob4* (Hu et al., 2016). At least three biological replicates were used for each condition.

## Supplementary Material

10.1242/develop.201376_sup1 Supplementary information

## References

[DEV201376C1] Altmann, M., Müller, P. P., Pelletier, J., Sonenberg, N. and Trachsel, H. (1989). A mammalian translation initiation factor can substitute for its yeast homologue in vivo. *J. Biol. Chem.* 264, 12145-12147. 10.1016/S0021-9258(18)63833-5 2663851

[DEV201376C2] Austin, C. M., Tan, M. H., Croft, L. J., Hammer, M. P. and Gan, H. M. (2015). Whole genome sequencing of the Asian Arowana (Scleropages formosus) provides insights into the evolution of ray-finned fishes. *Genome Biol. Evol.* 7, 2885-2895. 10.1093/gbe/evv186 26446539 PMC4684697

[DEV201376C3] Baek, M., Dimaio, F., Anishchenko, I., Dauparas, J., Ovchinnikov, S., Lee, G. R., Wang, J., Cong, Q., Kinch, L. N., Schaeffer, R. D. et al. (2021). Accurate prediction of protein structures and interactions using a 3-track neural network. *Science* 373, 871-876. 10.1126/science.abj8754 34282049 PMC7612213

[DEV201376C79] Bersell, K., Arab, S., Haring, B. and Kühn, B. (2009). Neuregulin1/ErbB4 signaling induces cardiomyocyte proliferation and repair of heart injury. *Cell* 138, 257-270. 10.1016/j.cell.2009.04.060 19632177

[DEV201376C4] Bezawork-Geleta, A., Rohlena, J., Dong, L., Pacak, K. and Neuzil, J. (2017). Mitochondrial complex II: at the crossroads. *Trends Biochem. Sci.* 42, 312-325. 10.1016/j.tibs.2017.01.003 28185716 PMC7441821

[DEV201376C5] Bian, C., Hu, Y., Ravi, V., Kuznetsova, I. S., Shen, X., Mu, X., Sun, Y., You, X., Li, J., Li, X. et al. (2016). The Asian arowana (Scleropages formosus) genome provides new insights into the evolution of an early lineage of teleosts. *Sci. Rep.* 6, 24501. 10.1038/srep24501 27089831 PMC4835728

[DEV201376C6] Borden, K. L. B. and Volpon, L. (2020). The diversity, plasticity, and adaptability of cap-dependent translation initiation and the associated machinery. *RNA Biol.* 17, 1-13. 10.1080/15476286.2020.1766179 32496897 PMC7549709

[DEV201376C7] Braasch, I., Gehrke, A. R., Smith, J. J., Kawasaki, K., Manousaki, T., Pasquier, J., Amores, A., Desvignes, T., Batzel, P., Catchen, J. et al. (2016). The spotted gar genome illuminates vertebrate evolution and facilitates human-teleost comparisons. *Nat. Genet.* 48, 427-437. 10.1038/ng.3526 26950095 PMC4817229

[DEV201376C80] Breinbauer, R. and Köhn, M. (2003). Azide–alkyne coupling: a powerful reaction for bioconjugate chemistry. *Chembiochem.* 4, 1147-1149. 10.1002/cbic.200300705 14613105

[DEV201376C8] Buccitelli, C. and Selbach, M. (2020). mRNAs, proteins and the emerging principles of gene expression control. *Nat. Rev. Genet.* 21, 630-644. 10.1038/s41576-020-0258-4 32709985

[DEV201376C9] Cardoso, A. C., Lam, N. T., Savla, J. J., Nakada, Y., Pereira, A. H. M., Elnwasany, A., Menendez-Montes, I., Ensley, E. L., Petric, U. B., Sharma, G. et al. (2020). Mitochondrial substrate utilization regulates cardiomyocyte cell-cycle progression. *Nat. Metab.* 2, 167-178. 10.1038/s42255-020-0169-x 32617517 PMC7331943

[DEV201376C10] Chen, B.-R., Wei, T.-W., Tang, C.-P., Sun, J.-T., Shan, T.-K., Fan, Y., Yang, T.-T., Li, Y.-F., Ma, Y., Wang, S.-B. et al. (2022). MNK2-eIF4E axis promotes cardiac repair in the infarcted mouse heart by activating cyclin D1. *J. Mol. Cell. Cardiol.* 166, 91-106. 10.1016/j.yjmcc.2022.02.006 35235835

[DEV201376C11] Chorghade, S., Seimetz, J., Emmons, R., Yang, J., Bresson, S. M., Lisio, M. D., Parise, G., Conrad, N. K. and Kalsotra, A. (2017). Poly(A) tail length regulates PABPC1 expression to tune translation in the heart. *eLife* 6, 568. 10.7554/eLife.24139 PMC5487213 28653618

[DEV201376C12] Davis, M. R., Delaleau, M. and Borden, K. L. B. (2019). Nuclear eIF4E stimulates 3′-end cleavage of target RNAs. *Cell Rep.* 27, 1397-1408.e4. 10.1016/j.celrep.2019.04.008 31042468 PMC6661904

[DEV201376C81] D'Uva, G., Aharonov, A., Lauriola, M., Kain, D., Yahalom-Ronen, Y., Carvalho, S., Weisinger, K., Bassat, E., Rajchman, D., Yifa, O. et al. (2015). ERBB2 triggers mammalian heart regeneration by promoting cardiomyocyte dedifferentiation and proliferation. *Nat. Cell Biol.* 17, 627-638. 10.1038/ncb3149 25848746

[DEV201376C13] Echtay, K. S., Roussel, D., St-Pierre, J., Jekabsons, M. B., Cadenas, S., Stuart, J. A., Harper, J. A., Roebuck, S. J., Morrison, A., Pickering, S. et al. (2002). Superoxide activates mitochondrial uncoupling proteins. *Nature* 415, 96-99. 10.1038/415096a 11780125

[DEV201376C14] Emmott, E., Jovanovic, M. and Slavov, N. (2018). Ribosome stoichiometry: from form to function. *Trends Biochem. Sci.* 44, 95-109. 10.1016/j.tibs.2018.10.009 30473427 PMC6340777

[DEV201376C15] Fang, Y., Gupta, V., Karra, R., Holdway, J. E., Kikuchi, K. and Poss, K. D. (2013). Translational profiling of cardiomyocytes identifies an early Jak1/Stat3 injury response required for zebrafish heart regeneration. *Proc. Natl. Acad. Sci. USA* 110, 13416-13421. 10.1073/pnas.1309810110 23901114 PMC3746860

[DEV201376C16] Fukuda, R., Marín-Juez, R., El-Sammak, H., Beisaw, A., Ramadass, R., Kuenne, C., Guenther, S., Konzer, A., Bhagwat, A. M., Graumann, J. et al. (2020). Stimulation of glycolysis promotes cardiomyocyte proliferation after injury in adult zebrafish. *EMBO Rep.* 21, e49752. 10.15252/embr.201949752 32648304 PMC7403660

[DEV201376C82] Gemberling, M., Karra, R., Dickson, A. L. and Poss, K. D. (2015). Nrg1 is an injury-induced cardiomyocyte mitogen for the endogenous heart regeneration program in zebrafish. *eLife* 4, e05871. 10.7554/eLife.05871 25830562 PMC4379493

[DEV201376C17] Genuth, N. R. and Barna, M. (2018). Heterogeneity and specialized functions of translation machinery: from genes to organisms. *Nat. Rev. Genet.* 19, 431-452. 10.1038/s41576-018-0008-z 29725087 PMC6813789

[DEV201376C18] Goldman, J. A., Kuzu, G., Lee, N., Karasik, J., Gemberling, M., Foglia, M. J., Karra, R., Dickson, A. L., Sun, F., Tolstorukov, M. Y. et al. (2017). Resolving heart regeneration by replacement histone profiling. *Dev. Cell* 40, 392-404.e5. 10.1016/j.devcel.2017.01.013 28245924 PMC5367476

[DEV201376C19] Graff, J. R., Konicek, B. W., Vincent, T. M., Lynch, R. L., Monteith, D., Weir, S. N., Schwier, P., Capen, A., Goode, R. L., Dowless, M. S. et al. (2007). Therapeutic suppression of translation initiation factor eIF4E expression reduces tumor growth without toxicity. *J. Clin. Invest.* 117, 2638-2648. 10.1172/JCI32044 17786246 PMC1957541

[DEV201376C20] Grüner, S., Peter, D., Weber, R., Wohlbold, L., Chung, M.-Y., Weichenrieder, O., Valkov, E., Igreja, C. and Izaurralde, E. (2016). The structures of eIF4E-eIF4G complexes reveal an extended interface to regulate translation initiation. *Mol. Cell* 64, 467-479. 10.1016/j.molcel.2016.09.020 27773676

[DEV201376C21] Harvey, S. A., Sealy, I., Kettleborough, R., Fenyes, F., White, R., Stemple, D. and Smith, J. C. (2013). Identification of the zebrafish maternal and paternal transcriptomes. *Development* 140, 2703-2710. 10.1242/dev.095091 23720042 PMC3678340

[DEV201376C22] Heinkele, F. J., Lou, B., Erben, V., Bennewitz, K., Poschet, G., Sticht, C. and Kroll, J. (2021). Metabolic and transcriptional adaptations improve physical performance of zebrafish. *Antioxidants (Basel)* 10, 1581. 10.3390/antiox10101581 34679716 PMC8533608

[DEV201376C23] Herberg, S., Gert, K. R., Schleiffer, A. and Pauli, A. (2018). The Ly6/uPAR protein Bouncer is necessary and sufficient for species-specific fertilization. *Science* 361, 1029-1033. 10.1126/science.aat7113 30190407 PMC6195191

[DEV201376C24] Hirose, K., Payumo, A. Y., Cutie, S., Hoang, A., Zhang, H., Guyot, R., Lunn, D., Bigley, R. B., Yu, H., Wang, J. et al. (2019). Evidence for hormonal control of heart regenerative capacity during endothermy acquisition. *Science* 364, 184-188. 10.1126/science.aar2038 30846611 PMC6541389

[DEV201376C25] Honkoop, H., de Bakker, D. E. M., Aharonov, A., Kruse, F., Shakked, A., Nguyen, P. D., de Heus, C., Garric, L., Muraro, M. J., Shoffner, A. et al. (2018). Single-cell analysis uncovers that metabolic reprogramming by ErbB2 signaling is essential for cardiomyocyte proliferation in the regenerating heart. *eLife* 8, e50163. 10.7554/eLife.50163 PMC7000220 31868166

[DEV201376C26] Hu, Y., Xie, S. and Yao, J. (2016). Identification of novel reference genes suitable for qRT-PCR normalization with respect to the zebrafish developmental stage. *PLoS ONE* 11, e0149277. 10.1371/journal.pone.0149277 26891128 PMC4758726

[DEV201376C27] Hu, B., Lelek, S., Spanjaard, B., El-Sammak, H., Simões, M. G., Mintcheva, J., Aliee, H., Schäfer, R., Meyer, A. M., Theis, F. et al. (2022). Origin and function of activated fibroblast states during zebrafish heart regeneration. *Nat. Genet.* 54, 1227-1237. 10.1038/s41588-022-01129-5 35864193 PMC7613248

[DEV201376C28] Huang, D. W., Sherman, B. T. and Lempicki, R. A. (2009). Systematic and integrative analysis of large gene lists using DAVID bioinformatics resources. *Nat. Protoc.* 4, 44-57. 10.1038/nprot.2008.211 19131956

[DEV201376C29] Ingolia, N. T., Brar, G. A., Rouskin, S., Mcgeachy, A. M. and Weissman, J. S. (2012). The ribosome profiling strategy for monitoring translation in vivo by deep sequencing of ribosome-protected mRNA fragments. *Nat. Protoc.* 7, 1534-1550. 10.1038/nprot.2012.086 22836135 PMC3535016

[DEV201376C30] Joshi, B., Lee, K., Maeder, D. L. and Jagus, R. (2005). Phylogenetic analysis of eIF4E-family members. *BMC Evol. Biol.* 5, 48. 10.1186/1471-2148-5-48 16191198 PMC1260017

[DEV201376C31] Jumper, J., Evans, R., Pritzel, A., Green, T., Figurnov, M., Ronneberger, O., Tunyasuvunakool, K., Bates, R., Žídek, A., Potapenko, A. et al. (2021). Highly accurate protein structure prediction with AlphaFold. *Nature* 596, 583-589. 10.1038/s41586-021-03819-2 34265844 PMC8371605

[DEV201376C32] Kang, J., Hu, J., Karra, R., Dickson, A. L., Tornini, V. A., Nachtrab, G., Gemberling, M., Goldman, J. A., Black, B. L. and Poss, K. D. (2016). Modulation of tissue repair by regeneration enhancer elements. *Nature* 532, 201-206. 10.1038/nature17644 27049946 PMC4844022

[DEV201376C33] Keegan, B. R., Feldman, J. L., Begemann, G., Ingham, P. W. and Yelon, D. (2005). Retinoic acid signaling restricts the cardiac progenitor pool. *Science* 307, 247-249. 10.1126/science.1101573 15653502

[DEV201376C34] Kikuchi, K., Holdway, J. E., Major, R. J., Blum, N., Dahn, R. D., Begemann, G. and Poss, K. D. (2011). Retinoic acid production by endocardium and epicardium is an injury response essential for zebrafish heart regeneration. *Dev. Cell* 20, 397-404. 10.1016/j.devcel.2011.01.010 21397850 PMC3071981

[DEV201376C35] Kong, J. and Lasko, P. (2012). Translational control in cellular and developmental processes. *Nat. Rev. Genet.* 13, 383-394. 10.1038/nrg3184 22568971

[DEV201376C36] Kubacka, D., Miguel, R. N., Minshall, N., Darzynkiewicz, E., Standart, N. and Zuberek, J. (2015). Distinct features of cap binding by eIF4E1b proteins. *J. Mol. Biol.* 427, 387-405. 10.1016/j.jmb.2014.11.009 25463438 PMC4306533

[DEV201376C37] Kusnadi, E. P., Timpone, C., Topisirovic, I., Larsson, O. and Furic, L. (2021). Regulation of gene expression via translational buffering. *Biochim. Biophys. Acta Mol. Cell Res.* 1869, 119140. 10.1016/j.bbamcr.2021.119140 34599983

[DEV201376C38] Lai, S.-L., Marín-Juez, R., Moura, P. L., Kuenne, C., Lai, J. K. H., Tsedeke, A. T., Guenther, S., Looso, M. and Stainier, D. Y. (2017). Reciprocal analyses in zebrafish and medaka reveal that harnessing the immune response promotes cardiac regeneration. *eLife* 6, 1382. 10.7554/eLife.25605 PMC5498136 28632131

[DEV201376C39] Lawrence, M., Huber, W., Pagès, H., Aboyoun, P., Carlson, M., Gentleman, R., Morgan, M. T. and Carey, V. J. (2013). Software for computing and annotating genomic ranges. *PLoS Comput. Biol.* 9, e1003118. 10.1371/journal.pcbi.1003118 23950696 PMC3738458

[DEV201376C40] Lee, H. J., Hou, Y., Chen, Y., Dailey, Z. Z., Riddihough, A., Jang, H. S., Wang, T. and Johnson, S. L. (2020). Regenerating zebrafish fin epigenome is characterized by stable lineage-specific DNA methylation and dynamic chromatin accessibility. *Genome Biol.* 21, 52. 10.1186/s13059-020-1948-0 32106888 PMC7047409

[DEV201376C41] Lepilina, A., Coon, A. N., Kikuchi, K., Holdway, J. E., Roberts, R. W., Burns, C. G. and Poss, K. D. (2006). A dynamic epicardial injury response supports progenitor cell activity during zebrafish heart regeneration. *Cell* 127, 607-619. 10.1016/j.cell.2006.08.052 17081981

[DEV201376C42] Lin, J.-J., Wang, F.-Y., Li, W.-H. and Wang, T.-Y. (2017). The rises and falls of opsin genes in 59 ray-finned fish genomes and their implications for environmental adaptation. *Sci. Rep.* 7, 15568. 10.1038/s41598-017-15868-7 29138475 PMC5686071

[DEV201376C43] Lorent, J., Kusnadi, E. P., van Hoef, V., Rebello, R. J., Leibovitch, M., Ristau, J., Chen, S., Lawrence, M. G., Szkop, K. J., Samreen, B. et al. (2019). Translational offsetting as a mode of estrogen receptor α–dependent regulation of gene expression. *EMBO J.* 38, e101323. 10.15252/embj.2018101323 31556460 PMC6885737

[DEV201376C44] Love, M. I., Huber, W. and Anders, S. (2014). Moderated estimation of fold change and dispersion for RNA-seq data with DESeq2. *Genome Biol.* 15, 550. 10.1186/s13059-014-0550-8 25516281 PMC4302049

[DEV201376C45] Ma, D., Tu, C., Sheng, Q., Yang, Y., Kan, Z., Guo, Y., Shyr, Y., Scott, I. C. and Lou, X. (2018). Dynamics of zebrafish heart regeneration using an HPLC–ESI–MS/MS approach. *J. Proteome Res.* 17, 1300-1308. 10.1021/acs.jproteome.7b00915 29369637

[DEV201376C46] Miklas, J. W., Levy, S., Hofsteen, P., Mex, D. I., Clark, E., Muster, J., Robitaille, A. M., Sivaram, G., Abell, L., Goodson, J. M. et al. (2021). Amino acid primed mTOR activity is essential for heart regeneration. *iScience* 25, 103574. 10.1016/j.isci.2021.103574 34988408 PMC8704488

[DEV201376C47] Minh, B. Q., Schmidt, H. A., Chernomor, O., Schrempf, D., Woodhams, M. D., von Haeseler, A. , and Lanfear, R. (2020). IQ-TREE 2: new models and efficient methods for phylogenetic inference in the genomic era. *Mol. Biol. Evol.* 37, 1530-1534. 10.1093/molbev/msaa015 32011700 PMC7182206

[DEV201376C48] Minshall, N., Reiter, M. H., Weil, D. and Standart, N. (2007). CPEB interacts with an ovary-specific eIF4E and 4E-T in early xenopus oocytes*. *J. Biol. Chem.* 282, 37389-37401. 10.1074/jbc.M704629200 17942399

[DEV201376C49] Mokalled, M. H., Patra, C., Dickson, A. L., Endo, T., Stainier, D. Y. R. and Poss, K. D. (2016). Injury-induced ctgfa directs glial bridging and spinal cord regeneration in zebrafish. *Science* 354, 630-634. 10.1126/science.aaf2679 27811277 PMC5114142

[DEV201376C50] Okuda, A. and Kimura, G. (1996). An amino acid change in novel protein D123 is responsible for temperature-sensitive G1-phase arrest in a mutant of rat fibroblast line 3Y1. *Exp. Cell Res.* 223, 242-249. 10.1006/excr.1996.0078 8601400

[DEV201376C51] Peri-Okonny, P. A., Baskin, K. K., Iwamoto, G., Mitchell, J. H., Smith, S. A., Kim, H. K., Szweda, L. I., Bassel-Duby, R., Fujikawa, T., Castorena, C. M. et al. (2019). High-phosphate diet induces exercise intolerance and impairs fatty acid metabolism in mice. *Circulation* 139, 1422-1434. 10.1161/CIRCULATIONAHA.118.037550 30612451 PMC6411426

[DEV201376C52] Perzlmaier, A. F., Richter, F. and Seufert, W. (2013). Translation initiation requires cell division cycle 123 (Cdc123) to facilitate biogenesis of the Eukaryotic Initiation Factor 2 (eIF2). *J. Biol. Chem.* 288, 21537-21546. 10.1074/jbc.M113.472290 23775072 PMC3724614

[DEV201376C53] Popa, A., Lebrigand, K., Paquet, A., Nottet, N., Robbe-Sermesant, K., Waldmann, R. and Barbry, P. (2016). RiboProfiling: a Bioconductor package for standard Ribo-seq pipeline processing. *F1000research* 5, 1309. 10.12688/f1000research.8964.1 27347386 PMC4918025

[DEV201376C54] Poss, K. D., Wilson, L. G. and Keating, M. T. (2002). Heart regeneration in zebrafish. *Science* 298, 2188-2190. 10.1126/science.1077857 12481136

[DEV201376C55] Pronobis, M. I., Zheng, S., Singh, S. P., Goldman, J. A. and Poss, K. D. (2021). In vivo proximity labeling identifies cardiomyocyte protein networks during zebrafish heart regeneration. *eLife* 10, e66079. 10.7554/eLife.66079 33764296 PMC8034980

[DEV201376C56] Robalino, J., Joshi, B., Fahrenkrug, S. C. and Jagus, R. (2004). Two zebrafish eIF4E family members are differentially expressed and functionally divergent. *J. Biol. Chem.* 279, 10532-10541. 10.1074/jbc.M313688200 14701818

[DEV201376C57] Sakurai, T., Tsuchida, M., Lampe, P. D. and Murakami, M. (2013). Cardiomyocyte FGF signaling is required for Cx43 phosphorylation and cardiac gap junction maintenance. *Exp. Cell Res.* 319, 2152-2165. 10.1016/j.yexcr.2013.05.022 23742896 PMC3783259

[DEV201376C58] Saraswathy, V. M., Zhou, L., Mcadow, A. R., Burris, B., Dogra, D., Reischauer, S. and Mokalled, M. H. (2022). Myostatin is a negative regulator of adult neurogenesis after spinal cord injury in zebrafish. *Cell Rep.* 41, 111705. 10.1016/j.celrep.2022.111705 36417881 PMC9742758

[DEV201376C59] Sénéchal, P., Robert, F., Cencic, R., Yanagiya, A., Chu, J., Sonenberg, N., Paquet, M. and Pelletier, J. (2021). Assessing eukaryotic initiation factor 4F subunit essentiality by CRISPR-induced gene ablation in the mouse. *Cell. Mol. Life Sci.* 78, 6709-6719. 10.1007/s00018-021-03940-5 34559254 PMC11073133

[DEV201376C60] Sherman, B. T., Hao, M., Qiu, J., Jiao, X., Baseler, M. W., Lane, H. C., Imamichi, T. and Chang, W. (2022). DAVID: a web server for functional enrichment analysis and functional annotation of gene lists (2021 update). *Nucleic Acids Res.* 50, W216-W221. 10.1093/nar/gkac194 35325185 PMC9252805

[DEV201376C61] Sosa, J. M., Huber, D. E., Welk, B. and Fraser, H. L. (2014). Development and application of MIPAR™: a novel software package for two- and three-dimensional microstructural characterization. *Integr. Mater. Manuf. Innovation* 3, 123-140. 10.1186/2193-9772-3-10

[DEV201376C62] Stockdale, W. T., Lemieux, M. E., Killen, A. C., Zhao, J., Hu, Z., Riepsaame, J., Hamilton, N., Kudoh, T., Riley, P. R., van Aerle, R. et al. (2018). Heart regeneration in the Mexican cavefish. *Cell Rep.* 25, 1997-2007.e7. 10.1016/j.celrep.2018.10.072 30462998 PMC6280125

[DEV201376C63] Stuart, T., Butler, A., Hoffman, P., Hafemeister, C., Papalexi, E., Mauck, W. M., Hao, Y., Stoeckius, M., Smibert, P. and Satija, R. (2019). Comprehensive integration of single-cell data. *Cell* 177, 1888-1902.e21. 10.1016/j.cell.2019.05.031 31178118 PMC6687398

[DEV201376C64] Sun, L., Gao, J., Dong, X., Liu, M., Li, D., Shi, X., Dong, J.-T., Lu, X., Liu, C. and Zhou, J. (2008). EB1 promotes Aurora-B kinase activity through blocking its inactivation by protein phosphatase 2A. *Proc. Natl. Acad. Sci. USA* 105, 7153-7158. 10.1073/pnas.0710018105 18477699 PMC2438220

[DEV201376C65] Sun, F., Ou, J., Shoffner, A. R., Luan, Y., Yang, H., Song, L., Safi, A., Cao, J., Yue, F., Crawford, G. E. et al. (2022). Enhancer selection dictates gene expression responses in remote organs during tissue regeneration. *Nat. Cell Biol.* 24, 685-696. 10.1038/s41556-022-00906-y 35513710 PMC9107506

[DEV201376C66] Syntichaki, P., Troulinaki, K. and Tavernarakis, N. (2007). eIF4E function in somatic cells modulates ageing in Caenorhabditis elegans. *Nature* 445, 922-926. 10.1038/nature05603 17277769

[DEV201376C67] Tahara, N., Akiyama, R., Wang, J., Kawakami, H., Bessho, Y. and Kawakami, Y. (2021). The FGF-AKT pathway is necessary for cardiomyocyte survival for heart regeneration in zebrafish. *Dev. Biol.* 472, 30-37. 10.1016/j.ydbio.2020.12.019 33444612 PMC7956161

[DEV201376C68] Taylor, J. S., Van de Peer, Y., Braasch, I. and Meyer, A. (2001). Comparative genomics provides evidence for an ancient genome duplication event in fish. *Philos. Trans. R. Soc. Lond. Ser. B Biol. Sci.* 356, 1661-1679. 10.1098/rstb.2001.0975 11604130 PMC1088543

[DEV201376C69] Thompson, J. D., Ou, J., Lee, N., Shin, K., Cigliola, V., Song, L., Crawford, G. E., Kang, J. and Poss, K. D. (2020). Identification and requirements of enhancers that direct gene expression during zebrafish fin regeneration. *Development* 147, dev191262. 10.1242/dev.191262 32665240 PMC7406312

[DEV201376C70] Trifinopoulos, J., Nguyen, L.-T., von Haeseler, A. , and Minh, B. Q. (2016). W-IQ-TREE: a fast online phylogenetic tool for maximum likelihood analysis. *Nucleic Acids Res.* 44, W232-W235. 10.1093/nar/gkw256 27084950 PMC4987875

[DEV201376C71] Truitt, M. L., Conn, C. S., Shi, Z., Pang, X., Tokuyasu, T., Coady, A. M., Seo, Y., Barna, M. and Ruggero, D. (2015). Differential requirements for eIF4E dose in normal development and cancer. *Cell* 162, 59-71. 10.1016/j.cell.2015.05.049 26095252 PMC4491046

[DEV201376C72] Wang, J., Panáková, D., Kikuchi, K., Holdway, J. E., Gemberling, M., Burris, J. S., Singh, S. P., Dickson, A. L., Lin, Y.-F., Sabeh, M. K. et al. (2011). The regenerative capacity of zebrafish reverses cardiac failure caused by genetic cardiomyocyte depletion. *Development* 138, 3421-3430. 10.1242/dev.068601 21752928 PMC3143562

[DEV201376C73] Wang, Z.-Y., Leushkin, E., Liechti, A., Ovchinnikova, S., Mößinger, K., Brüning, T., Rummel, C., Grützner, F., Cardoso-Moreira, M., Janich, P. et al. (2020a). Transcriptome and translatome co-evolution in mammals. *Nature* 588, 642-647. 10.1038/s41586-020-2899-z 33177713 PMC7116861

[DEV201376C74] Wang, W., Hu, C.-K., Zeng, A., Alegre, D., Hu, D., Gotting, K., Granillo, A. O., Wang, Y., Robb, S., Schnittker, R. et al. (2020b). Changes in regeneration-responsive enhancers shape regenerative capacities in vertebrates. *Science* 369, eaaz3090. 10.1126/science.aaz3090 32883834 PMC9479427

[DEV201376C75] White, R. J., Collins, J. E., Sealy, I. M., Wali, N., Dooley, C. M., Digby, Z., Stemple, D. L., Murphy, D. N., Billis, K., Hourlier, T. et al. (2017). A high-resolution mRNA expression time course of embryonic development in zebrafish. *eLife* 6, e30860. 10.7554/eLife.30860 29144233 PMC5690287

[DEV201376C76] Wu, C.-C., Kruse, F., Vasudevarao, M. D., Junker, J. P., Zebrowski, D. C., Fischer, K., Noël, E. S., Grün, D., Berezikov, E., Engel, F. B. et al. (2016). Spatially resolved genome-wide transcriptional profiling identifies Bmp signaling as essential regulator of zebrafish cardiomyocyte regeneration. *Dev. Cell* 36, 36-49. 10.1016/j.devcel.2015.12.010 26748692

[DEV201376C77] You, X., Bian, C., Zan, Q., Xu, X., Liu, X., Chen, J., Wang, J., Qiu, Y., Li, W., Zhang, X. et al. (2014). Mudskipper genomes provide insights into the terrestrial adaptation of amphibious fishes. *Nat. Commun.* 5, 5594. 10.1038/ncomms6594 25463417 PMC4268706

[DEV201376C78] Zhang, Y., Qin, C., Yang, L., Lu, R., Zhao, X. and Nie, G. (2018). A comparative genomics study of carbohydrate/glucose metabolic genes: from fish to mammals. *BMC Genomics* 19, 246. 10.1186/s12864-018-4647-4 29642853 PMC5896114

