# Supplementary material for: The translation initiation factor homolog eif4e1c regulates cardiomyocyte metabolism and proliferation during heart regeneration
Source: Development. 2023 Jun 12;150(20):dev201376. doi: 10.1242/dev.201376 (PMC10281269; doi:10.1242/dev.201376)
Supplement: Supplementary information [file develop-150-201376-s1.pdf]

Figure S1A

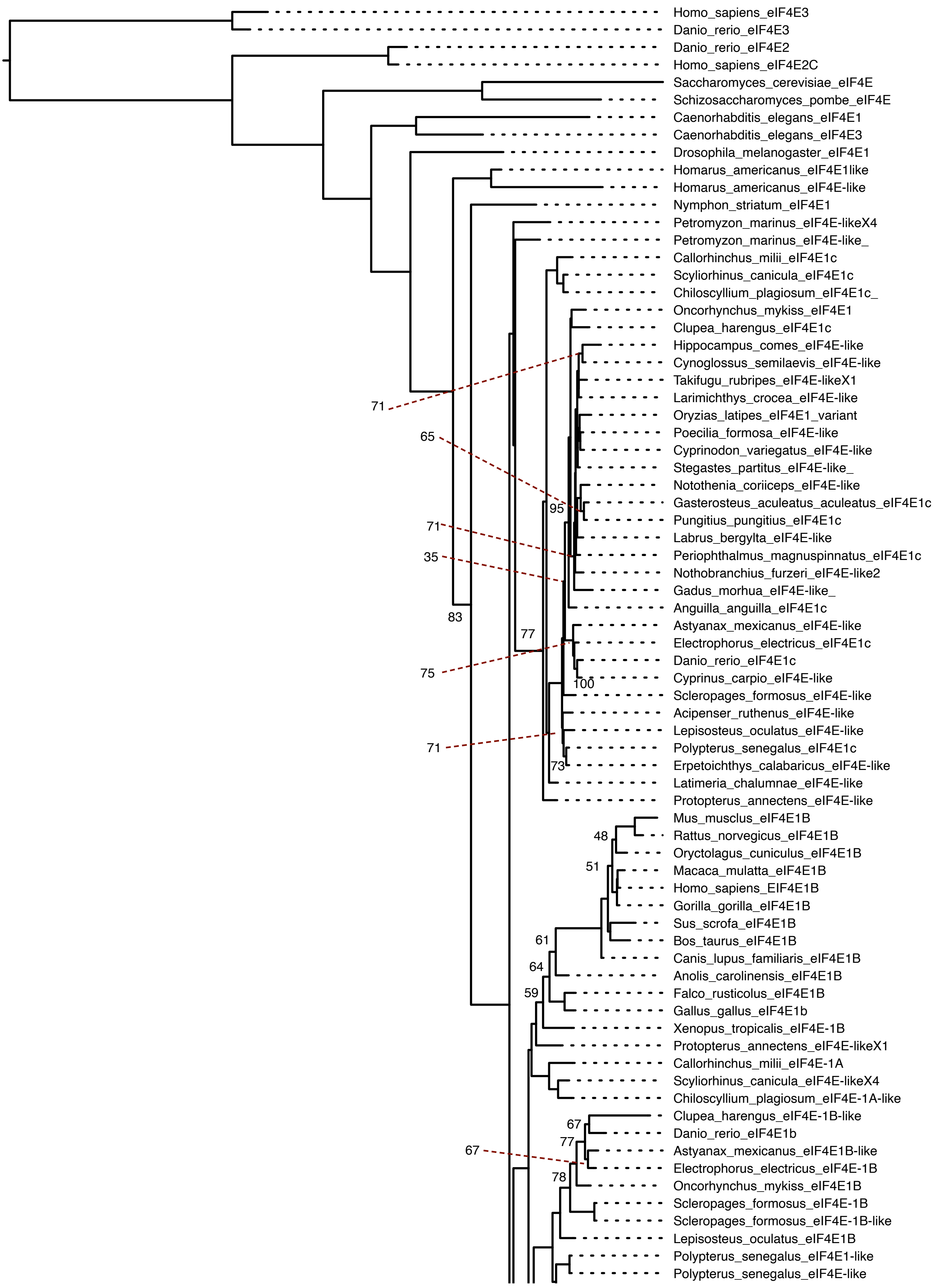

Development • Supplementary information

Figure S1A continued

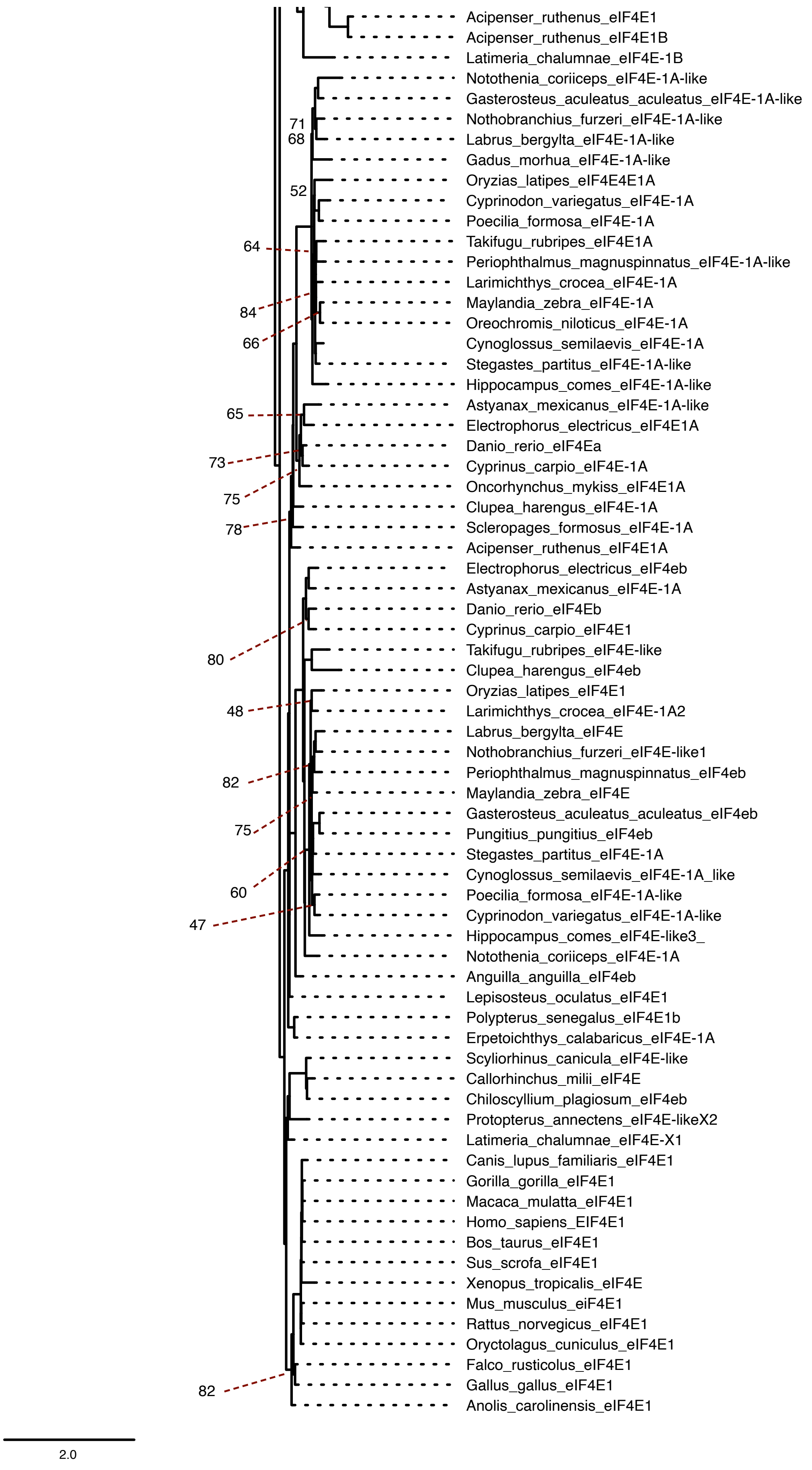

Development • Supplementary information

Figure S1B

```
Callorhinchus      NRWALWYFKNDKTKSWTENLRLIAKFDTVEDFWALYNHIQQPSKLLFGCDYCLFKDGIKP 103
Scyliorhinus       NRWALWYFKNDKTKGWTENLRLIAKFDTVEDFWALYNHIQQPSKLLFGCDYCLFKDGIKP 105
Chiloscyllium      NRWALWYFKNDKTKSWTENLRLIAKFDTVEDFWALYNHIQQPSKLLFGCDYCLFKDGIKP 105
Protopterus        NRWALWYFKNDKSKSWTENLRLIAKFDTVEDFWALYNHIQRPSKLQFGCDYCLFKDGIKP 99
Oncorhynchus       NKWALWYFKNDKSKSWTENLRLIAKFDTVEDFWALYNHIQQPSKLGFGCDYCLFKDGVKP 98
Oryzias            NRWALWYFKNDKTKSWTENLRLISKFDTVEDFWALYNHIQQPSKLVLGCDYCLFKDGIKP 94
Gadus              NKWALWYFKNDKSKSWTENLRLISKFDTVEDFWALYNHIQQPSKLGFGCDYCLFKDGIKP 93
Notothenia         NRWALWYFKNDKSKSWTENLRLISKFDTVEDFWALYNHIQQPSKLGFGCDYCLFKDGIKP 106
Anguilla           NRWALWYFKNDKSKSWTENLRLIAKFDTVEDFWALYNHIQQPSKLGYGCDYCLFKDGIKP 95
Gasterosteus       NRWALWYFKNDKSKSWTENLRLISKFDTVEDFWALYNHIQQPSKLGFGCDYSLFKDGIKP 92
Pungitius          NRWALWYFKNDKSKSWTENLRLISKFDTVEDFWALYNHIQQPSKLGYGCDYSLFKDGIKP 94
Nothobranchius     NRWALWYFKNDKSKSWTENLRLISKFDTVEDFWALYNHIQQPSKLNFGCDYCLFKDGIKP 93
Takifugu           NRWALWYFKNDKSKSWTENLRLISKFDTVEDFWALYNHIQQPSKLGFGCDYCLFKDGIKP 93
Labrus             NRWALWYFKNDKSKSWTENLRLISKFDTVEDFWALYNHIQQPSKLGFGCDYCLFKDGIKP 94
Cynoglossus        NKWALWYFKNDKSKSWTENLRLISKFDTVEDFWALYNHIQQPSKLGFGCDYCLFKDGIKP 91
Periophthalmus     NRWALWYFKNDKSKSWTENLRLISKFDTVEDFWALYNHIQQPSKLGFGCDYCLFKDGIKP 93
Poecilia           NRWALWYFKNDKSKSWTENLRLISKFDTVEDFWALYNHIQQPSKLGFGCDYSLFKDGIKP 93
Cyprinodon         NRWALWYFKNDKSKSWTENLRLISKFDTVEDFWALYNHIQQPSKLGYGCDYSLFKDGIKP 93
Stegastes          NRWALWYFKNDKSKSWTENLRLISKFDTVEDFWALYNHIQQPSKLGFGCDYCLFKDGIKP 93
Maylandia          NRWALWYFKNDKSKSWTENLRLISKFDTVEDFWALYNHIQQPSKLGFGCDYCLFKDGIKP 93
Oreochromis        NRWALWYFKNDKSKSWTENLRLISKFDTVEDFWALYNHIQQPSKLGFGCDYCLFKDGIKP 93
Larimichthys       NRWALWYFKNDKSKSWTENLRLISKFDTVEDFWALYNHIQQPSKLGFGCDYCLFKDGIKP 93
Latimeria          NKWALWYFKNDKSKSWTENLRLIAKFDTVEDFWALYNHIQQPSKLQFGCDYCLFKDGIKP 116
Clupea             NRWALWYFKNDKSKSWTENLRLIAKFDTVEDFWALYNHIQQPSKLGFGCDYCLFKDGIKP 117
Danio              NRWALWYFKNDKSKSWTENLRLISKFDTVEDFWALYNHIQQPSKLGFGCDYCLFKDGIKP 96
Cyprinus           NRWALWYFKNDKSKSWTENLRLISKFDTVEDFWALYNHIQQPSKLGFGCDYSLFKDGIKP 96
Astyanax           NRWALWYFKNDKSKSWTENLRLISKFDTVEDFWALYNHIQQPSKLGFGCDYCLFKDGIKP 114
Electrophorus      NRWALWYFKNDKSKSWTENLRLISKFDTVEDFWALYNHIQQPSKLGFGCDYCLFKDGIKP 95
Scleropages        NRWALWYFKNDKSKSWSENLRLIAKFDTVEDFWALYNHIQQPSKLGFGCDYCLFKDGIKP 95
Acipenser          NKWALWYFKNDKSKSWTENLRLIAKFDTVEDFWALYNHIQQPSKLGFGCDYCLFKDGIKP 95
Lepisosteus        NRWALWYFKNDKSKSWTENLRLIAKFDTVEDFWALYNHIQQPSKLGFGCDYCLFKDGIKP 95
Polypterus         NRWALWYFKNDKSKSWTENLRLIAKFDTVEDFWALYNHIQQPSKLGFGCDYCLFKDGIKP 95
Erpetoichthys      NRWALWYFKNDKSKSWTENLRLIAKFDTVEDFWALYNHIQQPSKLGFGCDYCLFKDGIKP 96
Hippocampus        NRWALWYFKNDKSKSWTENLRLISKFDTVEDFWALYNHIQQPSKLVYGCDYCLFKDGIKP 100
Petromyzon         NRWALWFYKNDKSKSWQANLRLITKVDTVEDFWALYNHIQVASRLMPGCDYSLFKDGIEP 102
                   *:****::****:* *  *****:*.**************  *.*  ****.*****::*
```

Sharks
Lungfish
Seahorse
Lamprey
Spotted Gar – recently diverged from teleost
Gray bichir – dragon eel – similar to lungfish
Mudskippers – technically teleost but use fins as legs
Reedfish – bichir – snake fish
Eel
Teleost

Development • Supplementary information

Figure S1B continued

```
Callorhinchus     MWEDDKNKKGGRWLMTLTKQQRHNDLDRYWLETLLCLIGEAFDEHSDEVCGAVVNVRPKG 163
Scyliorhinus      MWEDDKNKKGGRWLMTLNKQQRHNDLDRYWLETLLCLIGEAFDEHSDEVCGAVVNVRPKG 165
Chiloscyllium     MWEDDKNKRGGRWLMTLNKQQRHNDLDRYWLETLLCLIGEAFDEFSDEVCGAVVNVRPKG 165
Protopterus       MWEDELNKQGGRWLITLNKQQRHNDLDRYWLETLLCLIGEAFNDYSDDVCGAVVNVRPKG 159
Oncorhynchus      MWEDDKNKLGGRWLMTLSKQQRQIDLDRYWMETLLCLIGESFDEASEDVCGAVVNVRPKG 158
Oryzias           MWEDDKNKLGGRWLMTLNKQ-KHNDLDRYWMETLLCLVGESFDDASEEVCGAVVNVRHKG 153
Gadus             MWEDDRNKLGGRWLMTLNKQQRHNDLDRYWMETLLCLVGESFDESSEDVCGAVVNVRPKG 153
Notothenia        MWEDDRNKLGGRWLMTLNKQQRHNDLDRYWMETLLCLVGESFDAASEDVNGAVVNVRPKG 166
Anguilla          MWEDDRNKLGGRWLMTLNKQQRHNDLDRYWMETLLCLIGESFDEASDDVCGAVVNVRPKG 155
Gasterosteus      MWEDDRNKLGGRWLMTLNKQQRHNDLDRYWMETLLCLVGESFDEASEDVCGAVVNVRPKG 152
Pungitius         MWEDDRNKLGGRWLMTLNKQQRHNDLDRYWMETLLCLVGESFDEASEDVCGAVVNVRPKG 154
Nothobranchius    MWEDERNKLGGRWLITLNRQQRHNDLDRYWMETLLCLVGESFDEASDDVCGAVVNVRPKG 153
Takifugu          MWEDDRNKLGGRWLMTLNKQQRHNDLDRFWMETLLCLVGESFDEASDDVCGAVVNVRPKG 153
Labrus            MWEDDRNKLGGRWLMTLNKQQRHNDLDRYWMETLLCLVGESFDEASEDVCGAVVNVRPKG 154
Cynoglossus       MWEDDRNKLGGRWLMTLNKQQRHNDLDRYWMETLLCLVGESFDEASEDVCGAVVNVRPKG 151
Periophthalmus    MWEDDRNKLGGRWLMTLNKQQRHNDLDRYWMETLLCLVGESFDEASEDVCGAVVNVRPKG 153
Poecilia          MWEDDRNKLGGRWLMTLNKQQRHNDLDRYWMETLLCLVGESFDEASEEVCGAVVNVRPKG 153
Cyprinodon        MWEDDRNKLGGRWLMTLNKQQRHNDLDRYWMETLLCLVGDSFDEASEDVCGAVVNVRPKG 153
Stegastes         MWEDDRNKLGGRWLMTLNKQQRHNDLDRYWMETLLCLVGESFDEASEDVCGAVVNVRPKG 153
Maylandia         MWEDDRNKLGGRWLMTLNKQQRHNDLDRYWMETLLCLVGESFDEASEDVCGAVVNVRPKG 153
Oreochromis       MWEDDRNKLGGRWLMTLNKQQRHNDLDRYWMETLLCLVGESFDEASEDVCGAVVNVRPKG 153
Larimichthys      MWEDDRNKLGGRWLMTLNKQQRHNDLDRYWMETLLCLVGESFDEASEDVCGAVVNVRPKG 153
Latimeria         MWEDENNKRGGRWLMTLNKQQRHNDLDRYWLETLLCLIGESFDEHSDDVCGAVVNVRPKG 176
Clupea            MWEDDRNKLGGRWLMTLSKQQRHNDLDRYWMETLLCLIGESFDEASEDACGAVVNVRPKG 177
Danio             MWEDDRNKLGGRWLMTLSKQQRHNDLDRYWMETLLCLIGESFDEASEDVCGAVVNVRPKG 156
Cyprinus          MWEDDRNKLGGRWLMTLSKQQRHNDLDRYWMETLLCLIGESFDEASEDVCGAVVNVRPKG 156
Astyanax          MWEDDRNKLGGRWLMTLSKQQRHNDLDRYWMETLLCLIGESFDEASEDVCGAVVNVRPKG 174
Electrophorus     MWEDDRNKLGGRWLMTLNKQQRHNDLDRYWMETLLCLIGESFDEASEDVCGAVVNVRPKG 155
Scleropages       MWEDDRNKLGGRWLITLSKQQRHNDLDRYWMETLLCLIGESFDDASEDICGAVVNVRPKG 155
Acipenser         MWEDDRNKLGGRWLMTLNKQQRHNDLDRYWMETLLCLIGESFDEASEDVCGAVVNVRPKG 155
Lepisosteus       MWEDDRNKLGGRWLMTLGKQQRHNDLDRYWMETLLCLIGESFDEASDDVCGAVVNVRPKG 155
Polypterus        MWEDDRNKLGGRWLITLSKQQRHNDLDRYWMETLLCLIGESFDEASEDVCGAVVNVRPKG 155
Erpetoichthys     MWEDDRNKLGGRWLITLSKQQRHNDLDRYWMETLLCLIGESFDEASEDVCGAVVNVRPKG 156
Hippocampus       MWEDDRNKLGGRWLMTLNKVQRHNDLDRYWMETLLCLVGESFDEASDDVCGAVVNVRHKA 160
Petromyzon        MWEDERNKRGGRWLITLTKTQRHSDLDRYWLETLLCLIGEAFDDHSDDVCGAVVNVRPKA 162
                  ****: *: *****:** :  :: ****:*:******:*::*:  *::  ***:*** *.
```

Sharks
Lungfish
Seahorse
Lamprey
Spotted Gar – diverged from teleost
Gray bichir – dragon eel – appears lungfish
Mudskippers – technically teleost but use fins as legs
Reedfish – bichir – snake fish
Eel
Teleost

Development • Supplementary information

Development: doi:10.1242/dev.201376: Supplementary information

Figure S1B continued

```
Callorhinchus      DKISIWTGNCQSREAVTSIGQSYKERLGLPMKALIGYQSHDDTSSKSGSTTKNLYTV   220
Scyliorhinus       DKIAIWTGNCQNREAIVSIGQLYKERLGLSLKALIGYQSHDDTSSKSGSTTKNLFSV   222
Chiloscyllium      DKIAIWTGNCQNRDAVVSIGQLYKERLGLSLKALIGYQSHDDTSSKSGSTTKNLFSV   222
Protopterus        DKLSIWTTNCQNREAVVSIGQSYKERLGLPLKPVIGYQSHDDTSTKSGSTTKNLYSA   216
Oncorhynchus       DKISIWTGNCQNKEAIVAIGQQYKERLSIPIKLLIGYQSHDDTSSKSGSTTKNMYSV   215
Oryzias            DKISIWTGNCQNKEAIMTIGQLYKERLNLPMKAIIGYQSHDDTSSKSGSTTKNMYSV   210
Gadus              DKIAIWTSNCQNRDAIVTIGAGYKERLCLPSKPLISYQSHDDTSSKSGSTTKNMYSV   210
Notothenia         DKISIWTSNCQNRDAIMTIGQNYKERLSIPTKAIIGYQSHDDTSSKSGSTTKNIFSV   223
Anguilla           DKLAIWTGNCQNRDAIMTIGQQYKERLNIPSKALIGYQSHDDTSSKSGSTTKNMYTV   212
Gasterosteus       DKISIWTSQCQNRDAIMTIGQNYKERLNIPTKAIIGYQSHDDTSSKSGSTTKNMYSV   209
Pungitius          DKISIWTSQCQNRDAIMTIGQNYKERLNIPTKAIIGYQSHDDTSSKSGSTTKNMYSV   211
Nothobranchius     DKLAIWTSNCQNREAIMTIGQLYKERLSLPVKALIGYQSHDDTSSKSGSTTKNMYSV   210
Takifugu           DKIAIWTSNCQNREAIMTIGQLYKERLNIPIKAMLGYQSHDDTSSKSGSTTKNMYSI   210
Labrus             DKISIWTSNCQNRDAIMTIGQLYKERLTVPIKALIGYQSHDDTSSKSGSTTKNMYSV   211
Cynoglossus        DKIAIWTSNCQNREAIMTIGQQYKERLNIPIKAMIGYQSHDDTSSKSGSTTKNMYSV   208
Periophthalmus     DKIAIWTSNCQNRDAIMTIGQLYKERLSIPMKALIGYQSHDDTSSKSGSTTKNMYSV   210
Poecilia           DKISIWTSNCQNREAIMTIGQLYKERLNIPIKAMIGYQSHDDTSSKSGSTTKNMYSV   210
Cyprinodon         DKISIWTSNCQNREAIMTIGQLYKERLNLPIKAMIGYQSHDDTSSKSGSTTKNMYSV   210
Stegastes          DKIAIWTSNCQNREAIMTIGQLYKERLNLPIKAMIGYQSHDDTSSKSGSTTKNMYSV   210
Maylandia          DKISIWTSNCQNRDAIMTIGQLYKERLNLPMKAMIGYQSHDDTSSKSGSTTKNMYSV   210
Oreochromis        DKISIWTSNCQNRDAIMTIGQLYKERLNLPMKAMIGYQSHDDTSSKSGSTTKNMYSV   210
Larimichthys       DKIAIWTSNCQNRDAIMTIGQLYKERLNIPIKAMIGYQSHDDTSSKSGSTTKNMYSV   210
Latimeria          DKIAIWTTSCQNREAIMSIGQSYKERLGLPLKALIGYQSHDDTSSKSGSTTKNMYTV   233
Clupea             DKIAIWTANCQNRESIMTIGQQYKERLSIPNKTLIGYQSHDDTSSKSGSTTKNMYSV   234
Danio              DKIAIWTGNCQNRDAIMTIGQQYKERLSLPSKTLIGYQSHDDTSSKSGSTTKNMYSV   213
Cyprinus           DKISIWTGNCQNRDAIMTIGQQYKERLSLPIKTLIGYQSHDDTSSKSGSTTKNMYSV   213
Astyanax           DKIAIWTGNCQNRDAIMTIGLQYKERLNLPIKTLIGYQSHDDTSSKSGSTTKNMYSV   231
Electrophorus      DKIAIWTCNCQNRDAIMTIGQQYKERLNLPIKTLIGYQSHDDTSSKSGSTTKNMYSV   212
Scleropages        DKIAIWTVNCQNREAIMTIGQQYKERLNVPVKSLIGYQSHDDTSSKSGSTTKNMYTV   212
Acipenser          DKLAIWTGNCENKEAIMTIGQQYKERLSVPSKALIGYQSHDDTSSKSGSTTKNIFTV   212
Lepisosteus        DKISIWTGNCQNKEAIMTIGQQYKERLNVPNKALLGYQSHDDTSSKSGSTTKNMYTV   212
Polypterus         DKIAIWTGNCQNKEAIMTIGQLYKERLNMPLKALLGYQSHDDTSSKSGSTTKNMYTV   212
Erpetoichthys      DKIAIWTGNCQNKEAIMTIGQLYKERLNMPLKAILGYQSHDDTSSKSGSTTKNMYTV   213
Hippocampus        DKIAIWTSNCQNREAIMKIGETYKDLLSIPSKAMLGYQSHDDTSSKSGSTTKNMYSI   217
Petromyzon         DKIAVWTADCDNRESVVGIGRVYKDRLALPPRIIIGYQSHTDTATKSGSSTKNMFTV   219
                   **:::** . :.::::  **  **:** :  : ::.**** **::****:*** :
```

Sharks

Lungfish

Seahorse

Lamprey

Spotted Gar – diverged from teleost

Gray bichir – dragon eel – appears lungfish

Mudskippers – technically teleost but use fins as legs

Reedfish – bichir – snake fish

Eel

Teleost

Fig. S1. Phylogeny and conservation of eif4E orthologs.

(A) Shown is the phylogeny of Eif4e1c orthologues (all 53 species with 142 homologs) estimated using maximum likelihood using IQ-TREE. Nodes with bootstrap support < 0.85 are marked with their respective values, all other nodes had support values of 0.85 or higher. (B) ClustalW sequence alignment of Eif4e1c from aquatic vertebrate. Species names are color-coded by type with the legend on the bottom. Conserved Eif4e1c specific residues are highlighted in red bold with similar residues highlighted in pink.

Development • Supplementary information

Figure S2

A

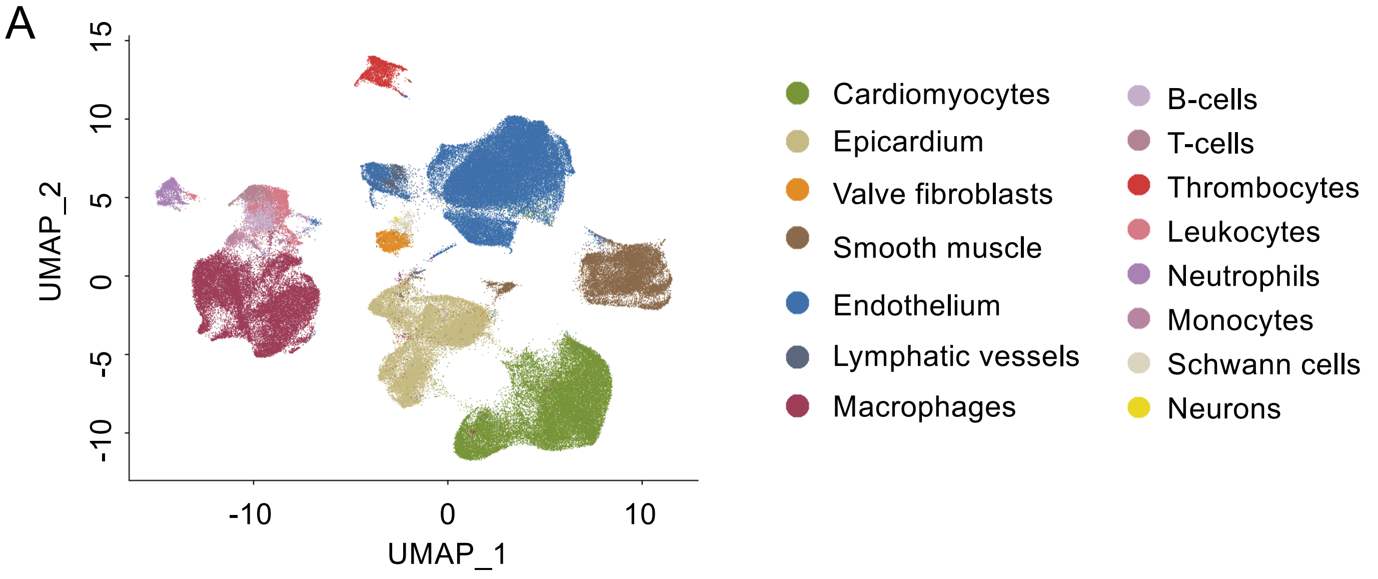

B

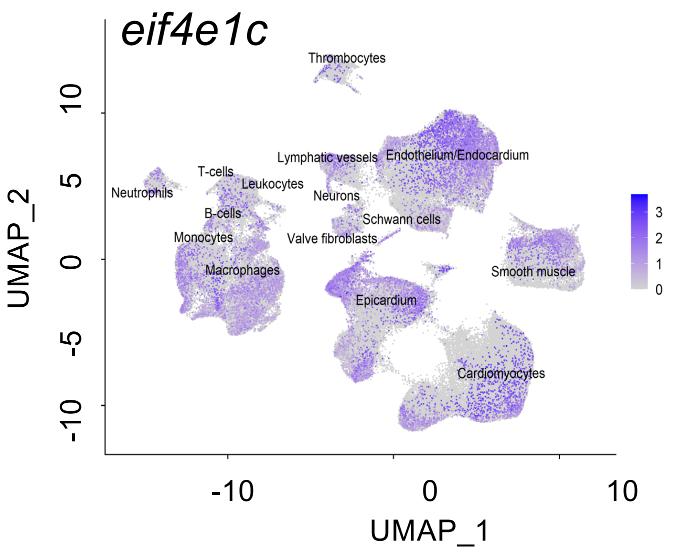

Cell-types	% *eif4e1c* (+) cells	% *eif4ea* (+) cells	% *eif4eb* (+) cells
Cardiomyocytes	4.58	1.45	4.47
Epicardium	17.51	7.98	14.46
Valve fibroblasts	7.49	3.39	10.97
Smooth muscle	9.56	5.50	9.86
Endothelium	10.64	4.44	7.27
Lymphatic vessels	12.86	4.36	8.18
Macrophages	13.67	4.53	5.36
B-cells	3.78	1.19	0.89
T-cells	1.94	0.91	0.67
Thrombocytes	2.69	0.96	1.69
Leukocytes	8.30	2.26	2.56
Neutrophils	5.89	2.26	2.39
Monocytes	11.49	6.42	3.54
Schwann cells	8.35	4.85	6.80
Neurons	42.86	14.29	2.04

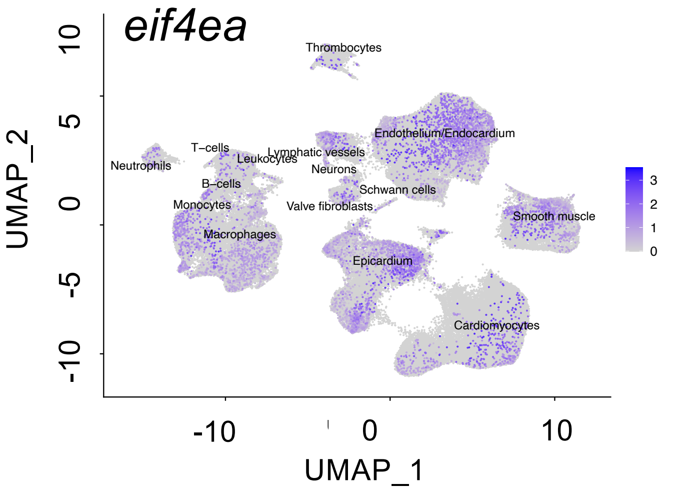

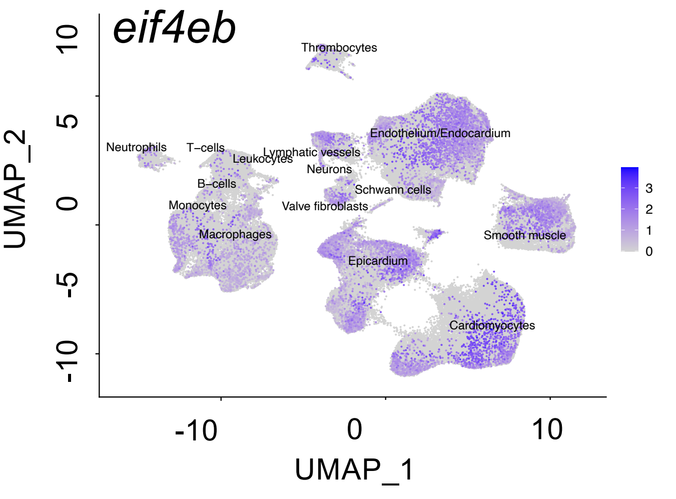

Development • Supplementary information

Fig. S2. EIF4E homologs are expressed in all cell-types within zebrafish hearts. (A) UMAP representation of single-cell RNA-seq data and clustering results of zebrafish hearts. (B) UMAP plots depicting *eif4e1c*, *eif4ea* and *eif4eb* expression. Right - Table showing the distribution of *eif4e1c* expressing cells in each cell clusters.

Development • Supplementary information

Figure S3

A

Cross	Stage	N	WT	HETS	HOMS	chi²	p value	Significant?
Δeif4e1c HETS X HETS	3dpf	190	51	85	54	1.94	0.33	no
	4wpf	410	109	203	98	0.629	0.73	no
	8wpf	630	207	312	111	29.3	4.31E-07	yes
	>3mpf	1868	599	951	318	84.9	3.22E-19	yes
Δeif4e1c HOMS ♀ X HETS ♂	3dpf	107	N/A	58	49	0.757	0.384	no

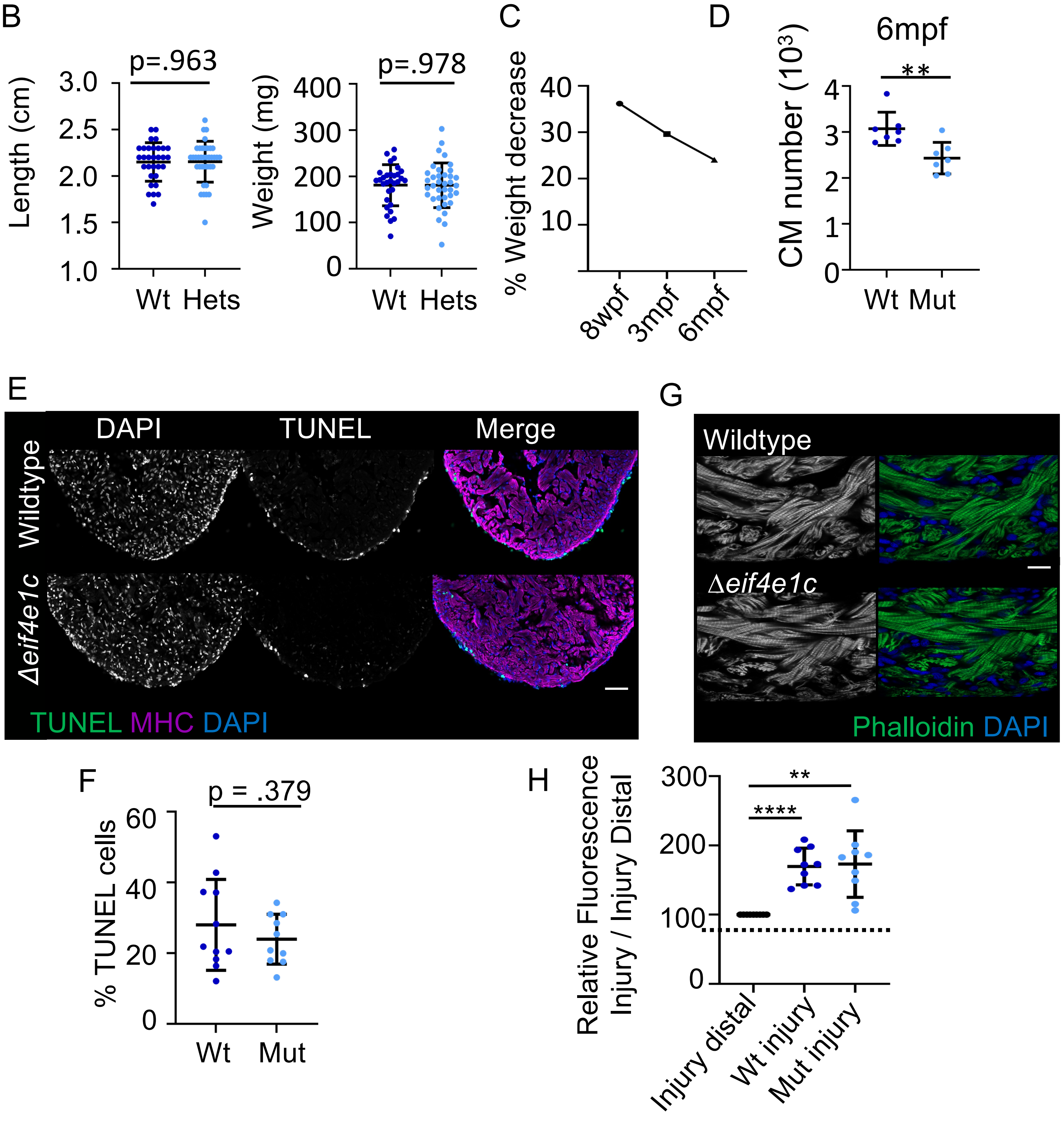

Development • Supplementary information

Fig. S3. Heterozygotes for De*if4e1c* were compared for length and weight with their wildtype siblings. (A) Table showing survival of progeny from crosses of Deif4e1c heterozygote carriers. Clutches of fish were genotyped at the listed development stage (dpf, wpf and mpf represent days-, weeks- or months-post-fertilization). Significant deviations from Mendelian ratios were calculated using chi^2 and the resulting p-value is shown. (B) Fish were grown together, genotyped at the listed stage (top label) and immediately measured from jaw to the caudal fin bifurcation (Average length WT= 2.15cm and mutant= 2.15cm with N = 31 vs 37; Welch's t-test, p-value =0.963,). After measuring length, fish were dried and weighed (Average weight: wildtype = 181.0mg and mutant = 180.7mg with N = 31 vs 37; Welch's t-test, p-value = 0.978,). (C) The percent weight decrease between mutant and wildtype fish was calculated for each of the time points (D) CM numbers for wildtype and De*if4e1c* mutants were counted at 6mpf from cryosections stained with an antibody for Mef2c (Scale bar = 100mm). The numbers of Mef2c positive cells were counted with MIPAR (average wildtype = 3070 and mutant = 2432 with N = 7 vs 7; Welch's t-test, p-value = 0.0055). (D) TUNEL staining of zebrafish hearts (Scale bar = 50mm). (F) Quantification of TUNEL stain showed no increase in the number of cells undergoing apoptosis in adult hearts (mean: wildtype = 28.03, mutant = 23.98; Welch's t-test, p-value = .379, N = 11 vs 10) (G) Phalloidin staining of wildtype (top) and mutant (bottom) zebrafish hearts. Left – Phalloidin in gray scale. Right – Merge of phalloidin with DAPI (Scale bar = 10mm). (H) Quantification of Eif4ea/b immunostaining from Figure 3G (wildtype mean increase (blue) = 1.70, Mann-Whitney p-value < 0.0001, N = 9 vs 9; mutant mean increase (light blue) = 1.73, Mann-Whitney p-value = 0.0018, N = 9 vs 9). Horizontal black bars display the mean (middle) or standard error (top and bottom).

Development • Supplementary information

Figure S4

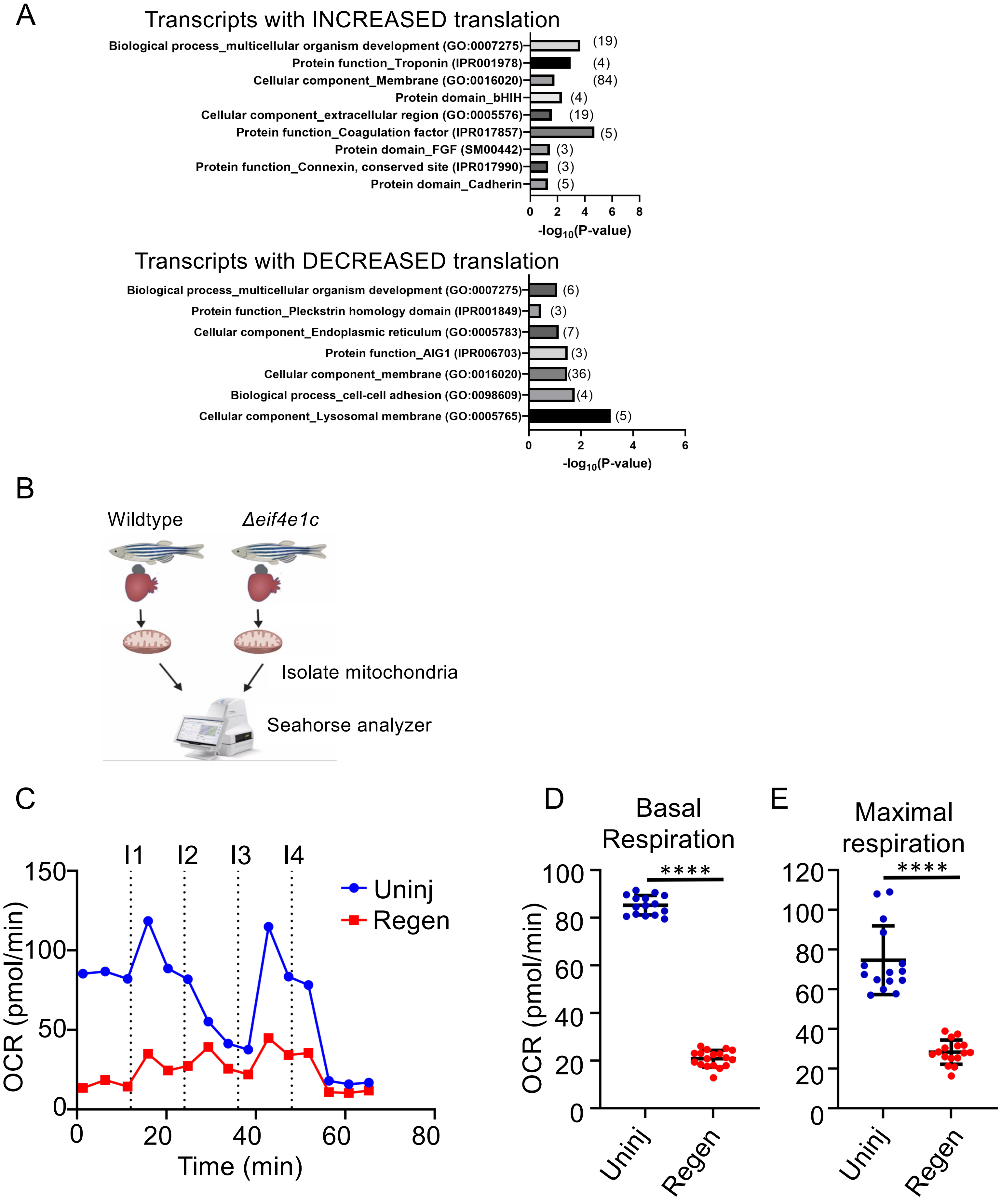

Development • Supplementary information

Fig. S4. (A) Gene ontology categories were discovered using the DAVID on-line platform. Each category is listed on the left with the identifier in parentheses. Shown are GO categories with p-value < 0.05, x-axis is $-\log_{10}$(p-value). Parentheses to the right of the bar indicate numbers of genes identified in each group. (B) Cartoon depicting experimental set-up for Seahorse analysis. Mitochondria were isolated from hearts dissected from wildtype and mutant fish and then processed on the Seahorse analyzer from Agilent. (C) Shown is a time-course of oxygen consumption rates (OCR) from mitochondria measured by the Seahorse analyzer. Hashed lines indicate time points of drug injection. The first injection (I1) is of ADP to stimulate respiration, the second injection (I2) is of oligomycin to inhibit ATP synthase (complex V) decreasing electron flow through transport chain, the third injection (I3) is of FCCP to uncouple the proton gradient, and the fourth injection (I4) is of antimycin A to inhibit complex III to shut down mitochondrial respiration. (D) Basal respiration is calculated as the OCR average after addition of ADP (I1 to I2) subtracting the non-mitochondrial respiration after injection of antimycin A (after I4) (mean: wildtype uninjured= 85.30 and wildtype regenerating= 20.90; Welch's t-test p value < 0.0001; N= 15,18). (E) Maximal respiration is calculated as the OCR average after FCCP addition (I3 to I4) subtracting the non-mitochondrial respiration after injection of antimycin A (after I4)(mean: wildtype uninjured= 74.64 and wildtype regenerating= 28.34; Mann-Whitney p-value < 0.0001; N= 15,18). For panels C-E, individual data points are represented by dots, uninjured is in blue, regenerating is in red, and horizontal black bars display the mean (middle) or standard error (top and bottom). Wildtype – Blue, Mutant – light blue.

Development • Supplementary information

Figure S5

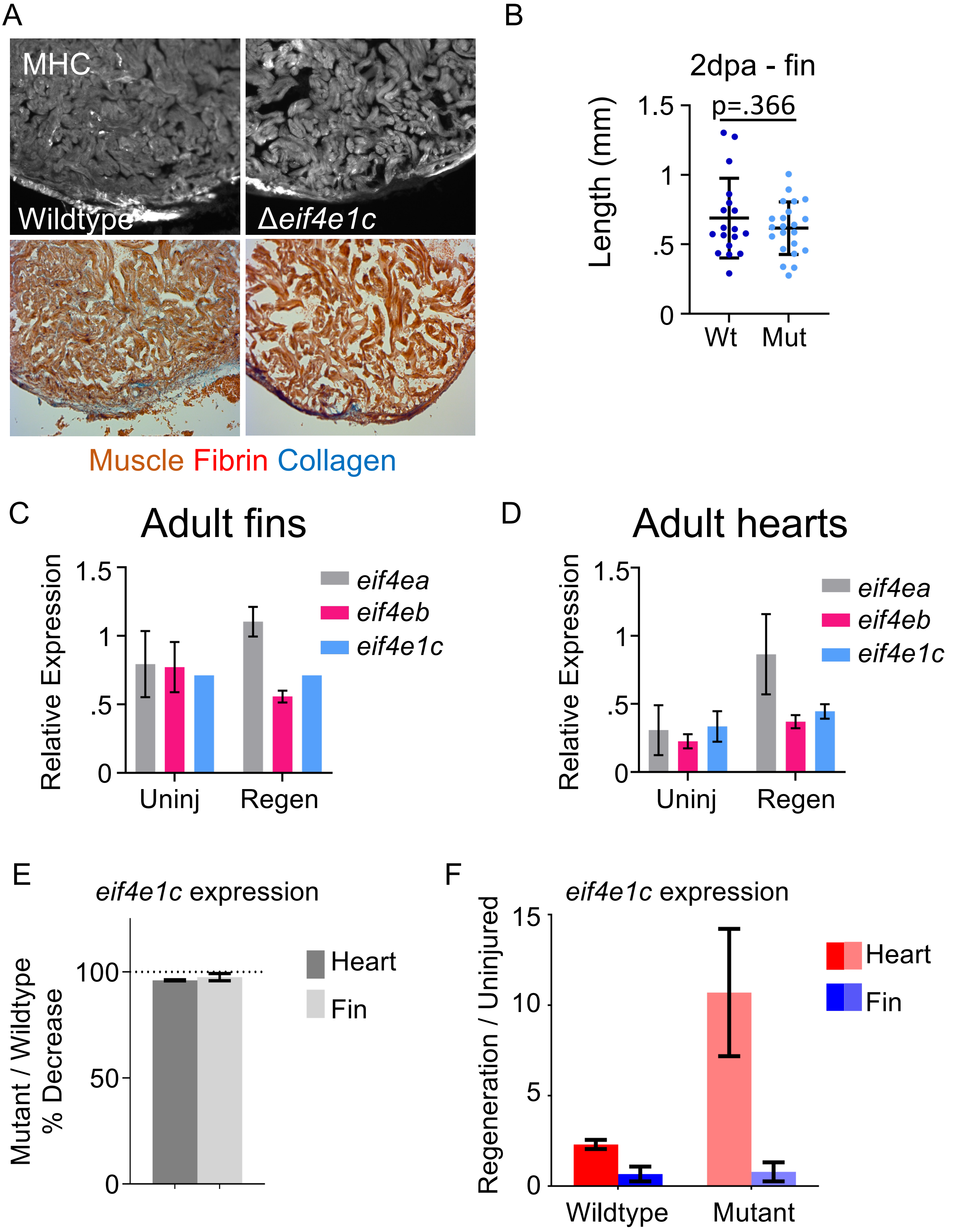

Development • Supplementary information

Fig. S5. (A) Images of sectioned ventricles at 28dpa are stained for Myosin heavy chain (MHC) to indicate cardiac muscle. Shown below are the same sections stained with AFOG which shows muscle (orange), Fibrin (red) and Collagen (blue). Hearts from 5 wildtype and 5 Δ*eif4e1c* mutants were ranked by injury size. Shown are representative hearts near the averages of the groups. (B) Caudal fins were amputated ~50% and the blastema were imaged 48 hours later for Δ*eif4e1c* mutants and their wildtype siblings. The length of the blastema was measured from the tip of the 3rd ray using the Zeiss microscope software. There was no significant difference between the groups (2dpa average wildtype = 0.689mm and mutant = 0.617mm; Welch's t-test, p-value = 0.366, N = 18 vs 22). Horizontal black bars display the mean (middle) or standard error (top and bottom). (C) Expression of each transcript was normalized to *mob4* in uninjured fins (left) and fins undergoing regeneration (right). (D) same as (C) but for the heart. (E) RT-qPCR analysis of *eif4e1c* from uninjured hearts (r dark gray) and fins (light gray) in Δ*eif4e1c* mutants. Ct values are normalized first to *mob4* and then to the wildtype values (hashed line). (F). RT-qPCR analysis of *eif4e1c* during regeneration of the heart (red) and the fin (blue). Wildtype is shown in dark colors (red and blue) and Δ*eif4e1c* mutants are shown in light colors (pink and light blue).

Figure S6

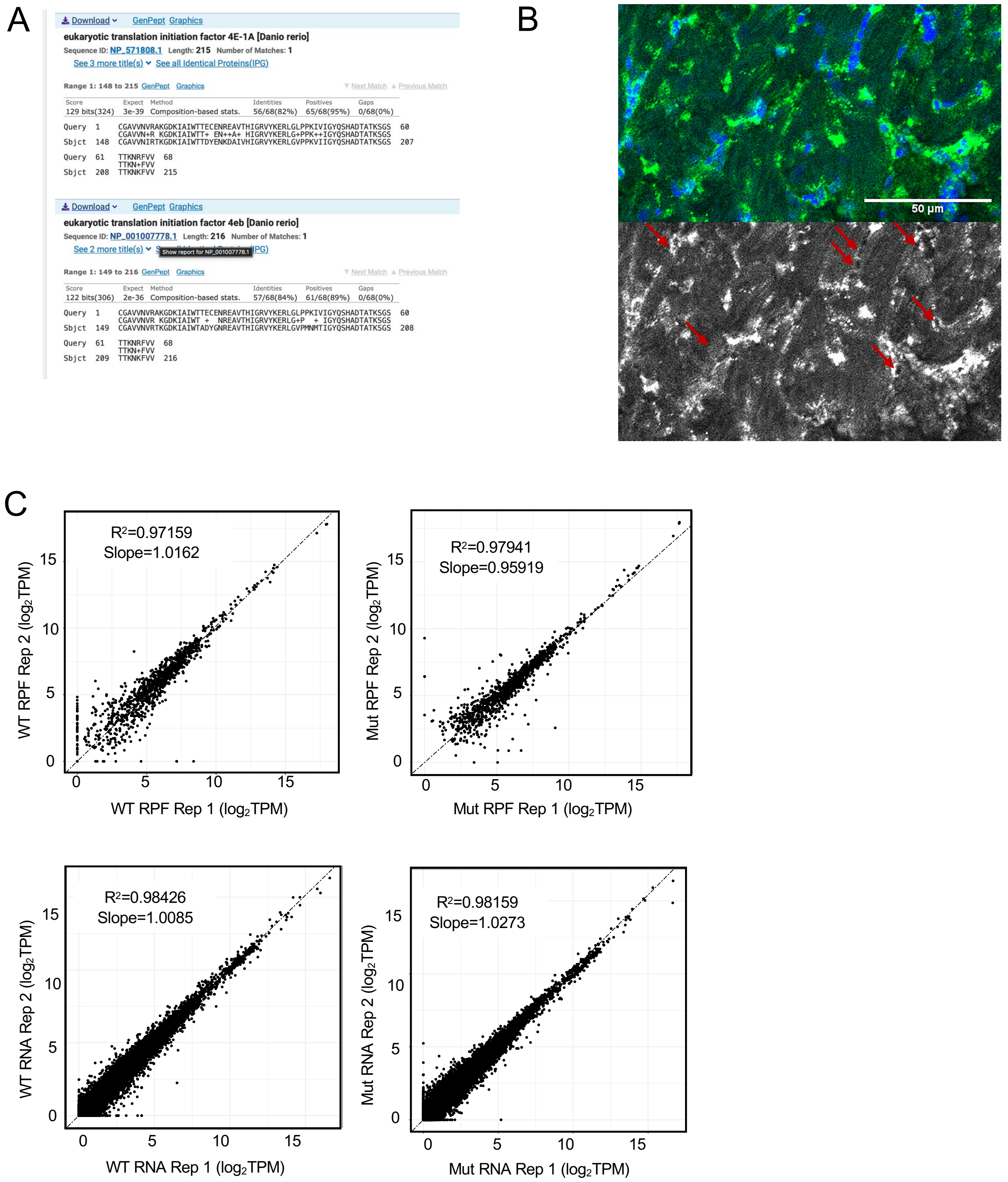

Fig. S6. (A) Region of human EIF4E1 (query) used to raise ab33768 is nearly identical in both paralogs of zebrafish canonical Eif4e1 (subject). (B) Immunofluorescence staining with ab33768 (green) on zebrafish hearts produces the expected cytoplasmic staining pattern (nuclei – blue). (C) Shown are the correlations between replicates for ribosome profiling (top) and RNA sequencing of the inputs (bottom) for both wildtype (left) and Δ*eif4e1c* mutant (right) samples.

Development • Supplementary information
