# Supplementary material for: The translation initiation factor homolog eif4e1c regulates cardiomyocyte metabolism and proliferation during heart regeneration
Source: Development. 2023 Jun 12;150(20):dev201376. doi: 10.1242/dev.201376 (PMC10281269; doi:10.1242/dev.201376)
Supplement: Supplementary file 1 [file develop-150-201376_review_history.pdf]

The translation initiation factor homolog *eif4e1c* regulates cardiomyocyte metabolism and proliferation during heart regeneration

Anupama Rao, Baken Lyu, Ishrat Jahan, Anna Lubertozzi, Gao Zhou, Frank Tedeschi, Eckhard Jankowsky, Junsu Kang, Bryan Carstens, Kenneth D. Poss, Kedryn Baskin and Joseph Aaron Goldman
DOI: 10.1242/dev.201376

Editor: Benoit Bruneau

Review timeline
Original submission: 19 October 2022
Editorial decision: 23 November 2022
First revision received: 17 March 2023
Accepted: 28 April 2023

Original submission

First decision letter

MS ID#: DEVELOP/2022/201376

MS TITLE: The Translation Initiation Factor Homolog, eif4e1c, Regulates Cardiomyocyte Metabolism and Proliferation During Heart Regeneration

AUTHORS: Anupama Rao, Baken Lyu, Anna Lubertozzi, Ishrat Jahan, Frank Tedeschi, Eckhard Jankowsky, Bryan Carstens, Kenneth Poss, Kedryn Baskin, and Joseph Aaron Goldman

I have now received all the referees reports on the above manuscript, and have reached a decision. The referees' comments are appended below, or you can access them online: please go to BenchPress and click on the 'Manuscripts with Decisions' queue in the Author Area.

The overall evaluation is positive and we would like to publish a revised manuscript in Development, provided that the referees' comments can be satisfactorily addressed. Please attend to all of the reviewers' comments in your revised manuscript and detail them in your point-by-point response. If you do not agree with any of their criticisms or suggestions explain clearly why this is so. If it would be helpful, you are welcome to contact us to discuss your revision in greater detail. Please send us a point-by-point response indicating your plans for addressing the referee's comments, and we will look over this and provide further guidance.

Reviewer 1

Advance summary and potential significance to field

The authors investigated a new elF4E1C family of the mRNA cap binding, translation initiation factor in heart development and regeneration from an evo-devo angle.
This is an interesting avenue and barely studied. They generated a new CRISPR mutant of eif4e1c to determine its requirement in zebrafish development.
Interestingly some eif4e1c mutants can survive to adults even though fewer homozygous mutants than expected based on the predicted Mendelian ratio were recovered after 8 weeks. Furthermore,

© 2023. Published by The Company of Biologists under the terms of the Creative Commons Attribution License (https://creativecommons.org/licenses/by/4.0/).

eif4e1c mutants have decreased cardiomyocyte numbers and impaired cardiomyocyte proliferation during heart regeneration.
However, fin regeneration is not affected in eif4e1c mutants, suggesting an organ specific role of eif4e1c. The authors performed translatome analysis and examined mitochondrial activity. Some of the data are less clear cut likely due to compensatory mechanisms and genetic compensation. These findings do not decrease the significance of this study and might even suggest interesting science underlying the roles of eilf4e1 factors.

Comments for the author

1. The authors should check e1f4e1c transcripts in eif4e1c mutants in embryos, at 4 weeks, and at 3 months. Is e1f4e1c mRNA degraded? If so, what other eif4e1 members can genetically compensate the loss of e1f4e1c?

2. It is interesting that eif4e1c mutants show specific heart phenotypes considering that both eif4e1aa and eif4e1ab are also expressed in the adult hearts. Is the idea that eif4e1c is cardiomyocyte specific or is the cardiomyocyte defect secondary to defects in other tissues? The authors should check expression of all three transcripts via in situ hybridization and RNAScope to determine if there is tissue/cell specificity. Furthermore, Is decreased cardiomyocyte number shown in Fig. 3E due to decreased cardiomyocyte proliferation?

3. The authors already observed decreased survival of eif4e1c at 4-8 wpf. Do the mutant hearts already start to show mutant phenotypes? Authors should include the cardiomyocyte quantification at 4 and 8 weeks in addition to 3 months or establish a time course when they first detect the difference in cardiomyocyte numbers. Furthermore, the authors should provide histology data in addition to the MHC staining, which does not reveal cardiomyocyte morphology at the current resolution. High resolution images are preferred.

4. The authors found that succinate dehydrogenase activity is slightly increased in mutants compared to controls during heart regeneration but not in injured hearts. Can the authors also check this during heart development to determine if this increased succinate dehydrogenase activity also accounts for the decreased cardiomyocyte numbers/proliferation?

5. There are no statistics for qRT-PCR data in Fig. S5 C and D. Furthermore what time point is for Fig. S5D (heart regeneration)? The data on the left do not seem to match the data on the right in Fig. S5D.

6. Minor comments.
    1. (Fig. S4A) in line 312—should be Fig. S5A.
    2. Images/panels in Fig. 1, Fig. 5B and Fig. 5C seem out of focus.
    3. Please specify what ZCAT stands for.

<u>Reviewer 2</u>

Advance summary and potential significance to field

In this interesting paper Rao et al., describe the presence of eIF4EC1 in the genome of all aquatic but not terrestrial species. They show that eIF4EC1 has homology to two other eIF4E family members known in zebrafish in critical regions important for cap-binding activity and interactions with eIF4G which is a bridging protein that brings in the ribosome to the 5Â’UTR of transcripts. Interestingly, eIF4EC1 has 23 amino acids characteristic of the eIF4EC1 family that are positioned mainly along the protein surface in solvent exposed regions, which may function in additional yet unknown protein-protein interactions. They characterize the expression of eIF4EC1 and find that it is widely expressed throughout development. Notably, eIF4EC1 expression is induced upon heart regeneration. Deletion of the eIF4EC1 gene locus causes poor growth and survival in adulthood. Ribosome profiling shows a discrete set of transcripts that are translationally regulated. They further show that deletion of eIF4EC1 impairs metabolic activity and regeneration in the heart. Together, this study sheds new light on the role of translational control in embryonic development and tissue regeneration. This is an important paper that is experimentally well carried out and will

© 2023. Published by The Company of Biologists under the terms of the Creative Commons Attribution License (https://creativecommons.org/licenses/by/4.0/).

significantly contribute to our understanding of a new layer of post-transcriptional control in development. I highly recommend the publication of this beautiful paper after some minor additional experiments that help to clarify the role of eIF4EC1 in translational control.

Comments for the author

Major Comments:

1) The authors use ribosome profiling to characterize translational changes in eIF4EC1 mutants. This is very informative, although it would be beautiful to do the same experiments upon heart regeneration. However, those would be more future experiments. What is instead very important is the knowledge of the role of eIF4EC1 on control of global protein synthesis which cannot be deduced by ribosome profiling. Therefore, a critical missing experiment is to carry out OP-Puromycin to quantify global protein synthesis in eIF4EC1 mutant embryos. It would be important to know whether eIF4EC1 regulates global and/or selective translational control.

2) The authors carry our ribosome profiling in eIF4EC1 mutants but they do not do any bioinformatics analysis to characterize whether there are any sequence motifs that might be enriched in the 5'UTRs of translationally regulated transcripts. There is growing evidence of transcript-specific translational control of eIF4E via the CERT, PRTE, or TOP-like sequences. Are any of these motifs enriched in the translationally regulated transcripts or does a new sequence motif emerge?

3) The authors often implicate compensation by other eIF4E family members in accounting for enhanced translation or the lack of a more complete regeneration phenotype that persists in the adult animals in eIF4EC1 mutants. However, there is a lack of direct measurements of eIF4EA or eIF4EB in these studies. The authors should examine what happens to the expression of these other eIF4E family members upon heart regeneration in eIF4EC1 mutants.

Reviewer 3

Advance summary and potential significance to field

In this study, the authors examine the role of the Translation Initiation Factor Homolog, eif4e1c in zebrafish heart development and regeneration. The authors show that the KO zebrafish have smaller size and smaller hearts, which appears to be secondary to decreased cardiomyocyte proliferation and cardiomyocyte number. The first two figures are somewhat thin showing basically the expression of Eif41A, B and C variants across species indicating that eif4e1c is only in aquatic animals.

Figure 2 shows the sequence conservation, the predicted structure (could have used alphafold here) and expression in various tissue. The last 3 figures basically show the effect of loss of function on heart development and regeneration and a tangential look at what happens mechanistically from a ribosomal profiling standpoint. There is also one panel where the overall metabolic profile of the mutant is examined.

Comments for the author

Overall this is a well written manuscript and the area is of interest. However at this stage these results fall short of fully describing the phenotype, or assessing the mechanism at enough depth to provide insights into how loss of function mediates cardiomyocyte growth and regeneration.

© 2023. Published by The Company of Biologists under the terms of the Creative Commons Attribution License (https://creativecommons.org/licenses/by/4.0/).

First revision

Author response to reviewers' comments

SUMMARY – We thank the reviewers for the time and consideration that they put into this manuscript. The reviewers appreciated the question and approach of our study. However, the reviewers asked for some further experiments to solidify our understanding. We have addressed each comment with either the experiments suggested or what we believe to be an equivalent. The manuscript is far better, and we thank the reviewers for their help.

We have also cleaned up the bioinformatics of our ribosome profiling. Some genes have been added and some lost but the overall points that we originally made have not changed. Categories uncovered by GO have changed though. We no longer see cell cycle factors enriched among the genes with decreased translational efficiency. However, some of the original cell cycle factors remain and we highlight these individually.

Below we address each of the reviewer comments individually:

Reviewer 1 Advance Summary and Potential Significance to Field:

The authors investigated a new elF4E1C family of the mRNA cap binding, translation initiation factor in heart development and regeneration from an evo-devo angle. This is an interesting avenue and barely studied. They generated a new CRISPR mutant of eif4e1c to determine its requirement in zebrafish development. Interestingly some eif4e1c mutants can survive to adults even though fewer homozygous mutants than expected based on the predicted Mendelian ratio were recovered after 8 weeks. Furthermore, eif4e1c mutants have decreased cardiomyocyte numbers and impaired cardiomyocyte proliferation during heart regeneration. However, fin regeneration is not affected in eif4e1c mutants, suggesting an organ specific role of eif4e1c. The authors performed translatome analysis and examined mitochondrial activity. Some of the data are less clear cut likely due to compensatory mechanisms and genetic compensation. These findings do not decrease the significance of this study and might even suggest interesting science underlying the roles of eilf4e1 factors.

Reviewer 1 Comments for the Author:

1. The authors should check *e1f4e1c* transcripts in *eif4e1c* mutants in embryos, at 4 weeks, and at 3 months. Is *e1f4e1c* mRNA degraded? If so, what other eif4e1 members can genetically compensate the loss of e1f4e1c?

As Reviewer 1 predicted, transcript levels of *eif4e1c* are 96% lower in mutants so we presume that the non-coding transcript is degraded (see original Fig. S4D). Interestingly, during regeneration *eif4e1c* transcript levels increase 12-fold in mutant hearts which is 6-fold higher than *eif4e1c* transcripts do in the wildtype. This suggests one mechanism of compensation is a feedback loop that stimulates *eif4e1c* transcription when Eif4e1c activity is absent. This only happens in the heart and not the fin further suggesting that Eif4e1c activity is more critical in the heart.

Please see revised manuscript:

Lines 365-376: "In uninjured hearts and fins, transcript levels for *eif4e1c* are reduced 96% and 97% from wildtype levels in the mutants (Fig. S5E). As expected, the mutant transcript retains the 3'UTR but is missing most of the coding sequence and is therefore likely degraded. Interestingly, *eif4e1c* transcript levels during heart regeneration increase 10.7-fold in mutants which is nearly 5-fold higher than *eif4e1c* increases in the wildtype hearts during regeneration (Fig. S5F, mean: wildtype = 2.30, mutant = 10.70). This suggests a mechanism of compensation where a feedback loop stimulates *eif4e1c* transcription when Eif4e1c activity is absent. Such a feedback loop does not exist in regenerating fins since there was no difference in the change of *eif4e1c* transcript abundance between wildtype and mutants (Fig. S5F, mean: wildtype = 0.667, mutant = 0.787). The existence of a feedback loop in the heart and not in the fin further argues that Eif4e1c activity is more critical to the heart during regeneration. We cannot exclude that other compensation mechanisms may mask the presence of this feedback loop in fins."

© 2023. Published by The Company of Biologists under the terms of the Creative Commons Attribution License (https://creativecommons.org/licenses/by/4.0/).

Reviewer 1 is also correct that other Eif4e1 family members are likely compensating for the loss of *eif4e1c*. Rather than solely look at transcript levels to probe mechanisms of compensation we used an antibody to detect the proteins. Originally raised against the human form of canonical EIF4E, the antibody (ab33768) targets a region containing long stretches of amino acids that are identical between both zebrafish paralogs and the human form (95% similar and 82% identical, see new Fig. S6A). By western blot the antibody detects a band at the expected migration of 25kD (see new Fig. 3F) and by immunofluorescence the antibody demonstrates the expected cytoplasmic staining pattern in zebrafish hearts suggesting that it recognizes zebrafish canonical Eif4e1 (see new Fig. S6B). With this antibody we now report that canonical Eif4e1 protein levels increase 1.8-fold in *eif4e1c* mutant hearts. Thus, one likely mechanism to partially compensate for Eif4e1c loss is increased canonical protein levels.

Please see revised manuscript:

Lines 225-237: "Similarities in sequence and expression patterns between *eif4e1c* and its canonical homologs *eif4ea* and *eif4eb* raises the possibility that D*eif4e1c* mutants survive because the canonical homologs can functionally substitute. To look at canonical protein levels, we used an antibody raised against the human canonical EIF4E1 that also recognizes the zebrafish orthologs (see Methods for details). Western blot of whole cell extracts from wildtype and D*eif4e1c* mutant hearts showed that canonical Eif4ea/b protein levels are increased in D*eif4e1c* mutants (Fig. 3F, fold change avg. = 1.82, N = 4,4). Interestingly, we see Eif4ea/b protein levels increase at the site of injury during wildtype heart regeneration (Fig. 3G). In both wildtype and D*eif4e1c* mutant hearts, after amputation of the apex of the ventricle, Eif4ea/b protein levels increase to a similar extent at the site of injury (Fig. S3H). Taken together, canonical eIF4E1 protein levels increase in D*eif4e1c* mutant hearts, as they do during wildtype heart regeneration. We conclude that canonical Eif4ea/b likely partially compensates for cardiac growth deficits in surviving D*eif4e1c* mutant hearts."

2. It is interesting that eif4e1c mutants show specific heart phenotypes considering that both eif4e1aa and eif4e1ab are also expressed in adult hearts. Is the idea that eif4e1c is cardiomyocyte specific or is the cardiomyocyte defect secondary to defects in other tissues?

We do not know whether our observed cardiac phenotypes are a result of defects specific to cardiomyocytes or due to defects in other cell-types. This is an important question to answer. To formally demonstrate whether defects in cell numbers are autonomous to CMs we are currently constructing a transgenic line expressing Eif4e1c in CMs. We predict that the transgene will rescue the mutant phenotype because Eif4e1c is functioning directly in CMs. These experiments are 6-8 months away and we hope to report an interesting result in the future. In this manuscript we now more carefully word our statements to make clear that we do not yet know from which cell-type the defect is arising.

Please see revised manuscript:

Lines 279-283: "We cannot formally determine from which cell-types these translational changes are occurring. Yet, we predict at least some changes are occurring in CMs since CMs compromise most cells in uninjured zebrafish hearts and decreased translation of cell proliferation transcripts like *mapre1a* and *cdc123* are likely occurring there.

Lines 332-333: "We cannot determine if *eif4e1c* proliferation phenotypes are CM specific or a result of secondary effects from other cell-types."

3. The authors should check the expression of all three transcripts via in situ hybridization and RNAScope to determine if there is tissue/cell specificity.

RNA Scope can visualize cell-type specificity; however, in our experience, high levels of background of RNA Scope on muscle in zebrafish hearts make robust analysis challenging. Other cell-types are more readily observed cleanly (see Gemberling et al. eLife 2015;4:e05871). Instead, to address the cell-type-specificity of *eif4e1c,* we analyzed several published single-cell RNA-seq data sets from zebrafish hearts (Hu et al., 2022). Transcripts for *eif4e1c* were detected in nearly every single

© 2023. Published by The Company of Biologists under the terms of the Creative Commons Attribution License (https://creativecommons.org/licenses/by/4.0/).

cluster of cell-types including in cardiomyocytes (see new Fig. S2). We now report in the manuscript that *eif4e1c* (and the two canonical paralogs) are widely expressed in all major cell-types of the heart.

Please see revised manuscript:

Lines 150-156: “While *eif4e1c* expression is widespread, whether it is confined to a particular cell-types within organs is unclear. We analyzed a published scRNAseq data set produced from adult zebrafish hearts that uncovered 15 different identifiable cell-types (Hu et al., 2022). Transcripts for *eif4e1c,* and the canonical *eif4ea* and *eif4eb*, were detected within each of the 15 clusters suggesting that all three transcripts are expressed in all cardiac cell-types including in CMs (Fig. S2). Thus, like its canonical orthologs, *eif4e1c* is broadly expressed in all organ systems and cell-types examined.”

4. Furthermore, is decreased cardiomyocyte number shown in Fig. 3E due to decreased cardiomyocyte proliferation?

The reviewer makes a very good point. We cannot formally say whether decreased CM numbers are a result of reduced CM proliferation, reduced CM survival, or increased CM death. To address this point further we carried out TUNEL staining on *eif4e1c* mutant hearts and wildtype siblings to measure levels of apoptosis and observed no detectable increase in apoptosis in mutants of *eif4e1c* (see new Fig. S3E). We have also modified our language to make clear that we cannot formally state that the developmental phenotype of reduced numbers of CMs could result from reduced CM-proliferation *or* from reduced CM-survival.

Please see revised manuscript:

Lines 215-220: “Reduced CM numbers may result from less CM proliferation, reduced CM survival, or increased CM death. To see if hearts from D*eif4e1c* mutant fish were undergoing increased apoptosis, we performed TUNEL on heart sections from mutant and wildtype fish and observed no significant change (Fig. S3E-F). We conclude that deficits in overall growth of adult *eif4e1c* mutants likely reflect defects in either cell proliferation or cell survival during development.”

5. The authors already observed decreased survival of eif4e1c at 4-8 wpf. Do the mutant hearts already start to show mutant phenotypes? Authors should include the cardiomyocyte quantification at 4 and 8 weeks in addition to 3 months or establish a time course when they first detect the difference in cardiomyocyte numbers.

We agree with the reviewer that it is important to see if the phenotypes of reduced cardiac growth and increased death of *eif4e1c* mutants coincide. However, a time course of counting CMs would only give a result that is correlative. A direct connection between impaired CM growth and mutant death would still be lacking. To directly answer the question, we are planning to express Eif4e1c only in CMs to see if such a transgene would rescue both phenotypes. However, this experiment is 6-9 months away and we believe is beyond the scope of this paper. To address the authors concern, we now include an earlier timepoint and count CMs in *eif4e1c* mutants at 8-weeks post-fertilization (see new Fig. 3E).

Please see revised manuscript:

Lines 211-213: “Fewer CMs were also measured at 8wpf just after mutant death occurred (Fig. 3E, mean: wildtype = 1078, mutant = 848, p-value = 0.0388, n=17, 17)."

6. Furthermore, the authors should provide histology data in addition to the MHC staining, which does not reveal cardiomyocyte morphology at the current resolution. High resolution images are preferred.

We agree with the reviewer that it is important to see if CM morphology changes in the mutant. It is possible that an altered morphology underlies the impairment to proliferate. To address this point, we used phalloidin-488 (Abcam – ab176753), which binds to filamentous actin found in sarcomeres. In the revised manuscript we include images of mutant and wildtype CMs stained with

© 2023. Published by The Company of Biologists under the terms of the Creative Commons Attribution License (https://creativecommons.org/licenses/by/4.0/).

phalloidin (new Fig. S3G). For imaging, we used a 63X objective rather than the 10X objective we used for the MHC staining. There is no difference in structure observed between mutants and wildtype CMs.

Please see revised manuscript:
Lines 220-223: "Phalloidin staining of sarcomeres revealed no gross differences between CM sarcomere structure in wildtype and mutant hearts from (Fig. S3G). Likely, proliferation or survival deficits are unrelated to structural differences in D*eif4e1c* mutant CMs."

7. The authors found that succinate dehydrogenase activity is slightly increased in mutants compared to controls during heart regeneration but not in injured hearts. Can the authors also check this during heart development to determine if this increased succinate dehydrogenase activity also accounts for the decreased cardiomyocyte numbers/proliferation?

The defect in succinate dehydrogenase activity appears to be regeneration specific since there is no difference between uninjured adult hearts (Fig. 5C and 5D). We now include a line indicating that these conditions are not significantly different. This was missing from the original submission, and we thank the reviewer for bringing this to our attention. We do not expect that this result will change at the earlier timepoints.

Moreover, the resolution of the succinate dehydrogenase assay makes it difficult to see differences between individual CMs that might be occurring at 8wpf. Our ability to detect differences during regeneration are largely based on the organ-wide injury model (ZCAT). High resolution comparisons between adjacent CMs are not required to detect differences in these conditions.

8. There are no statistics for qRT-PCR data in Fig. S5 C and D. Furthermore, what time point is for Fig. S5D (heart regeneration)? The data on the left do not seem to match the data on the right in Fig. S5D.

Thank you for bringing this to our attention. The Reviewer was correct to question the reliability of a single biological replicate for qPCR. As an alternative we turned to published RNAseq profiles. We used multiple biological replicates sometimes from the same paper and where possible from different published manuscripts.

Please see revised manuscript:
Lines 144-150: "To determine if *eif4e1c* is expressed in all tissues like eIF4E1A or is restricted to discrete organs or developmental stages like *eif4e1b*, we surveyed expression of *eif4e1c* in published RNAseq data sets (see Methods). Each of *eif4ea, eif4eb,* and *eif4e1c* genes were expressed in every organ examined (Fig. 2C). Expression of *eif4e1c* was highest in the fin and in other tissues ranged from 38% to 67% of that total. These differences are likely within the typical RNAseq variability due to batch effects, so we conclude that *eif4e1c* is widely expressed at similar levels throughout zebrafish organ systems."

The original data in Fig. S5C (left) with S5D (right) did not match because on was from fin regeneration and the other from heart regeneration. We do not expect that they will match. Now our figures are more clearly labelled. Please see new Fig. S5CD.

9. Minor comments.
1.(Fig. S4A) in line 312—should be Fig. S5A.

Thank you for identifying this typo. Some figure labeling has changed in this new submission, but we have tried to take better care to make sure the text is accurate. Our apologies.

2. Images/panels in Fig. 1, Fig. 5B and Fig. 5C seem out of focus.

We have now replaced these images with higher resolution versions.

3. Please specify what ZCAT stands for.
Thank you for bringing this to our attention.

© 2023. Published by The Company of Biologists under the terms of the Creative Commons Attribution License (https://creativecommons.org/licenses/by/4.0/).

Please see revised manuscript:
Lines 342: “Using the zebrafish cardiomyocyte ablation transgenes (ZCAT), we injured CMs…”

Reviewer 2 Advance Summary and Potential Significance to Field:

In this interesting paper Rao et al., describe the presence of eIF4EC1 in the genome of all aquatic but not terrestrial species. They show that eIF4EC1 has homology to two other eIF4E family members known in zebrafish in critical regions important for cap-binding activity and interactions with eIF4G which is a bridging protein that brings in the ribosome to the 5’UTR of transcripts. Interestingly, eIF4EC1 has 23 amino acids characteristic of the eIF4EC1 family that are positioned mainly along the protein surface in solvent exposed regions, which may function in additional yet unknown protein-protein interactions. They characterize the expression of eIF4EC1 and find that it is widely expressed throughout development. Notably, eIF4EC1 expression is induced upon heart regeneration. Deletion of the eIF4EC1 gene locus causes poor growth and survival in adulthood. Ribosome profiling shows a discrete set of transcripts that are translationally regulated. They further show that deletion of eIF4EC1 impairs metabolic activity and regeneration in the heart. Together, this study sheds new light on the role of translational control in embryonic development and tissue regeneration. This is an important paper that is experimentally well carried out and will significantly contribute to our understanding of a new layer of post-transcriptional control in development. I highly recommend the publication of this beautiful paper after some minor additional experiments that help to clarify the role of eIF4EC1 in translational control.

Reviewer 2 Comments for the Author:
Major Comments:
1. The authors use ribosome profiling to characterize translational changes in eIF4EC1 mutants. This is very informative, although it would be beautiful to do the same experiments upon heart regeneration. However, those would be more future experiments.

We thank the reviewer for this comment. We agree that it would be very interesting to check translational changes occurring in *eif4e1c* mutant hearts during heart regeneration and intend to examine this in a future study.

What is instead very important is the knowledge of the role of eIF4EC1 on control of global protein synthesis which cannot be deduced by ribosome profiling. Therefore, a critical missing experiment is to carry out OP- Puromycin to quantify global protein synthesis in eIF4EC1 mutant embryos. It would be important to know whether eIF4EC1 regulates global and/or selective translational control.

We thank the reviewer for suggesting this experiment. We carried out the OPP assay to examine global protein synthesis in uninjured WT and *eif4e1c* mutant hearts (see new Fig. 4AB). There is no change in average global protein synthesis in hearts of surviving *eif4e1c* mutants.

Please see revised manuscript:

Lines 249-2526: “To measure global translation in *eif4e1c* mutants we injected fish with O-propargyl-puromycin (OPP), which terminates peptide chain elongation. Total translation (incorporation of OPP) can be measured by fluorescence levels by conjugating fluorophores using CLIC chemistry (Fig. 4A). Injection of OPP into D*eif4e1c* mutants and wildtype siblings demonstrated that global protein synthesis is unperturbed in D*eif4e1c* mutants (Fig. 4B; mean fluorescence: wildtype = 140279 adu/sq.µm, mutant = 140596 adu/sq.µm; p-value = 0.806, Mann-Whitney, N = 15,15). We conclude that growth deficits in D*eif4e1c* mutants are not a result of impaired general translation.”

The authors carry our ribosome profiling in eIF4EC1 mutants but they do not do any bioinformatics analysis to characterize whether there are any sequence motifs that might be enriched in the 5’UTRs of translationally regulated transcripts. There is growing evidence of transcript-specific translational control of eIF4E via the CERT, PRTE, or TOP-like sequences.

© 2023. Published by The Company of Biologists under the terms of the Creative Commons Attribution License (https://creativecommons.org/licenses/by/4.0/).

Are any of these motifs enriched in the translationally regulated transcripts or does a new sequence motif emerge?

We agree with the reviewer that motif analysis of our ribosome profiling experiment could be very interesting. We carried out MEME analysis of 5' and 3' UTRs of the genes that are translated more and translated less in the uninjured eif4e1c mutant hearts. Unfortunately, our analysis did not reveal motifs that were specific to the coding strand of the mRNA, so we believe that the uncovered motifs are likely artifacts. For this reason, we do not include this data within the paper.

The ribosome profiling does not reveal direct targets of Eif4e1c. Moreover, we believe that compensation is happening in the *eif4e1c* mutants by the canonical factors Eif4ea/b (see question 3 below). Compensation likely masks many direct Eif4e1c targets that would improve our MEME analysis. We will revisit using MEME to find discrete motifs when we have an improved list of Eif4e1c targets.

3. The authors often implicate compensation by other eIF4E family members in accounting for enhanced translation or the lack of a more complete regeneration phenotype that persists in the adult animals in eIF4EC1 mutants. However, there is a lack of direct measurements of eIF4EA or eIF4EB in these studies. The authors should examine what happens to the expression of these other eIF4E family members upon heart regeneration in eIF4EC1 mutants.

We have tried to address this concern by carrying out Western blot of canonical Eif4e1a in uninjured WT and eif4e1c mutant hearts. Our data showed that the levels of canonical proteins increase in *eif4e1c* mutant hearts (see new Fig. 3F). Likely, there is partial compensation by canonical orthologs in surviving *eif4e1c* mutants. We also examine canonical protein levels during regeneration and see canonical factors increasing to a similar degree (see new Fig.3G).

Please see revised manuscript:

Lines 224-237: “In contrast to reported *Eif4e1a* mutants in mice, zebrafish *eif4e1c* deletion knockouts can survive (Altmann et al., 1989; Sénéchal et al., 2021). Similarities in sequence and expression patterns between *eif4e1c* and its canonical homologs *eif4ea* and *eif4eb* raises the possibility that D*eif4e1c* mutants survive because the canonical homologs can functionally substitute. To look at canonical protein levels, we used an antibody raised against the human canonical EIF4E1 that also recognizes the zebrafish orthologs (see Methods for details). Western blot of whole cell extracts from wildtype and D*eif4e1c* mutant hearts showed that canonical Eif4ea/b protein levels are increased in D*eif4e1c* mutants (Fig. 3F, fold change avg. = 1.82, N = 4,4). Interestingly, we see Eif4ea/b protein levels increase at the site of injury during wildtype heart regeneration (Fig. 3G). In both wildtype and D*eif4e1c* mutant hearts, after amputation of the apex of the ventricle, Eif4ea/b protein levels increase to a similar extent at the site of injury (Fig. S3H). Taken together, canonical eIF4E1 protein levels increase in D*eif4e1c* mutant hearts, as they do during wildtype heart regeneration. We conclude that canonical Eif4ea/b likely partially compensates for cardiac growth deficits in surviving D*eif4e1c* mutant hearts.”

Please also see Reviewer 1, Point 1 for further discussion of compensation mechanisms beyond the canonical proteins.

Reviewer 3 Advance Summary and Potential Significance to Field:

In this study, the authors examine the role of the Translation Initiation Factor Homolog, eif4e1c in zebrafish heart development and regeneration. The authors show that the KO zebrafish have smaller size and smaller hearts, which appears to be secondary to decreased cardiomyocyte proliferation and cardiomyocyte number. The first two figures are somewhat thin showing basically the expression of Eif41A, B and C variants across species indicating that eif4e1c is only in aquatic animals.

Figure 2 shows the sequence conservation, the predicted structure (could have used alpha fold here) and expression in various tissue. The last 3 figures basically show the effect of loss of function on heart development and regeneration and a tangential look at what happens

© 2023. Published by The Company of Biologists under the terms of the Creative Commons Attribution License (https://creativecommons.org/licenses/by/4.0/).

mechanistically from a ribosomal profiling standpoint. There is also one panel where the overall metabolic profile of the mutant is examined.

Reviewer 3 Comments for the Author:

Overall this is a well written manuscript and the area is of interest. However at this stage these results fall short of fully describing the phenotype, or assessing the mechanism at enough depth to provide insights into how loss of function mediates cardiomyocyte growth and regeneration.

We thank the reviewer for the comment that the manuscript is interesting and well written. We understand the frustration that a full understanding of the mutant phenotype remains unclear. Hopefully, more light will be shed on mechanisms for Eif4e1c during heart development and regeneration in future studies.

Respectfully, we have a philosophical disagreement with the reviewer that a full understanding of mutant phenotypes should be a pre-requisite for publication. If a manuscript is novel, interesting, rigorous, and adds to scientific knowledge then it should be shared with the community. Mechanisms of many critical regulatory pathways from Polycomb repression to Shh signaling gradients (and dozens more) are taking decades to unravel after their initial reporting. They are still being debated in manuscripts with high visibility and a clear consensus on mechanism is lacking after hundreds if not thousands of manuscripts. There are many examples from many fields where incomplete understanding of mechanism did not impair progress. For example, in the disease spinal muscular atrophy there are excellent therapies but the critical pathways downstream of SMN deficiency remain an enigma.

Burying data for 10-20 years in a scientific notebook until a full accounting is possible does not help further knowledge as much as sharing data with the community. Practically, such a requirement would extend training periods far beyond reason and there is no funding mechanism that would entertain such a timeline.

Also, we note that we mentioned in the first submission that Eif4e1c is identical to canonical proteins by Alphafold (please see line 133 in resubmission). However, cap- binding proteins change their orientation when bound to their functional substrates, a 5'methyl cap and EIF4G. Therefore, we used the more relevant structure to model the highly conserved 23 amino acids rather than rely on alphafold.

Second decision letter

MS ID#: DEVELOP/2022/201376

MS TITLE: The Translation Initiation Factor Homolog, eif4e1c, Regulates Cardiomyocyte Metabolism and Proliferation During Heart Regeneration

AUTHORS: Anupama Rao, Baken Lyu, Ishrat Jahan, Anna Lubertozzi, Gao Zhou, Frank Tedeschi, Eckhard Jankowsky, Junsu Kang, Bryan Carstens, Kenneth Poss, Kedryn Baskin, and Joseph Aaron Goldman
ARTICLE TYPE: Research Article

Apologies for the delay, we never received one of the reviewer comments despite multiple reqminder. I am happy to tell you that your manuscript has been accepted for publication in Development, pending our standard ethics checks.

© 2023. Published by The Company of Biologists under the terms of the Creative Commons Attribution License (https://creativecommons.org/licenses/by/4.0/).

Reviewer 1

Advance summary and potential significance to field

The authors investigated a new elF4E1C family of the mRNA cap binding translation initiation factor in heart development and regeneration from an evo-devo angle. This is an interesting avenue and understudied. They generated a new CRISPR mutant of eif4e1c to determine its requirement in zebrafish development.

Interestingly some eif4e1c mutants can survive to adults even though fewer homozygous mutants than expected based on the predicted Mendelian ratio were recovered after 8 weeks. Furthermore, eif4e1c mutants have decreased cardiomyocyte numbers and impaired cardiomyocyte proliferation during heart regeneration. However, fin regeneration is not affected in eif4e1c mutants suggesting an organ specific role of eif4e1c. Mechanistically, the authors performed translatome analysis and examined mitochondrial activity. Their findings are significance and suggest important functions of eilf4e1 factors.

Comments for the author

The authors have addressed my previous concerns. Some of the suggested experiments might take long time to perform and they can pursue these experiments in the future.

© 2023. Published by The Company of Biologists under the terms of the Creative Commons Attribution License (https://creativecommons.org/licenses/by/4.0/).
